# Spectral-temporal processing of naturalistic sounds in monkeys and humans

**DOI:** 10.1152/jn.00129.2023

**Published:** 2023-11-15

**Authors:** Robert F. van der Willigen, Huib Versnel, A. John van Opstal

**Affiliations:** ^1^Section Neurophysics, Donders Institute for Brain, Cognition and Behaviour, Radboud University, Nijmegen, The Netherlands; ^2^School of Communication, Media and Information Technology, Rotterdam University of Applied Sciences, Rotterdam, The Netherlands; ^3^Research Center Creating 010, Rotterdam University of Applied Sciences, Rotterdam, The Netherlands; ^4^Department of Otorhinolaryngology and Head & Neck Surgery, UMC Utrecht Brain Center, University Medical Center Utrecht, Utrecht University, Utrecht, The Netherlands

**Keywords:** naturalistic sounds, primate audition, psychophysics, spectrotemporal modulation transfer functions, spectrum-time separability

## Abstract

Human speech and vocalizations in animals are rich in joint spectrotemporal (S-T) modulations, wherein acoustic changes in both frequency and time are functionally related. In principle, the primate auditory system could process these complex dynamic sounds based on either an inseparable representation of S-T features or, alternatively, a separable representation. The separability hypothesis implies an independent processing of spectral and temporal modulations. We collected comparative data on the S-T hearing sensitivity in humans and macaque monkeys to a wide range of broadband dynamic spectrotemporal ripple stimuli employing a yes-no signal-detection task. Ripples were systematically varied, as a function of density (spectral modulation frequency), velocity (temporal modulation frequency), or modulation depth, to cover a listener’s full S-T modulation sensitivity, derived from a total of 87 psychometric ripple detection curves. Audiograms were measured to control for normal hearing. Determined were hearing thresholds, reaction time distributions, and S-T modulation transfer functions (MTFs), both at the ripple detection thresholds and at suprathreshold modulation depths. Our psychophysically derived MTFs are consistent with the hypothesis that both monkeys and humans employ analogous perceptual strategies: S-T acoustic information is primarily processed separable. Singular value decomposition (SVD), however, revealed a small, but consistent, inseparable spectral-temporal interaction. Finally, SVD analysis of the known visual spatiotemporal contrast sensitivity function (CSF) highlights that human vision is space-time inseparable to a much larger extent than is the case for S-T sensitivity in hearing. Thus, the specificity with which the primate brain encodes natural sounds appears to be less strict than is required to adequately deal with natural images.

**NEW & NOTEWORTHY** We provide comparative data on primate audition of naturalistic sounds comprising hearing thresholds, reaction time distributions, and spectral-temporal modulation transfer functions. Our psychophysical experiments demonstrate that auditory information is primarily processed in a spectral-temporal-independent manner by both monkeys and humans. Singular value decomposition of known visual spatiotemporal contrast sensitivity, in comparison to our auditory spectral-temporal sensitivity, revealed a striking contrast in how the brain encodes natural sounds as opposed to natural images, as vision appears to be space-time inseparable.

## Graphical abstract

**Figure:**
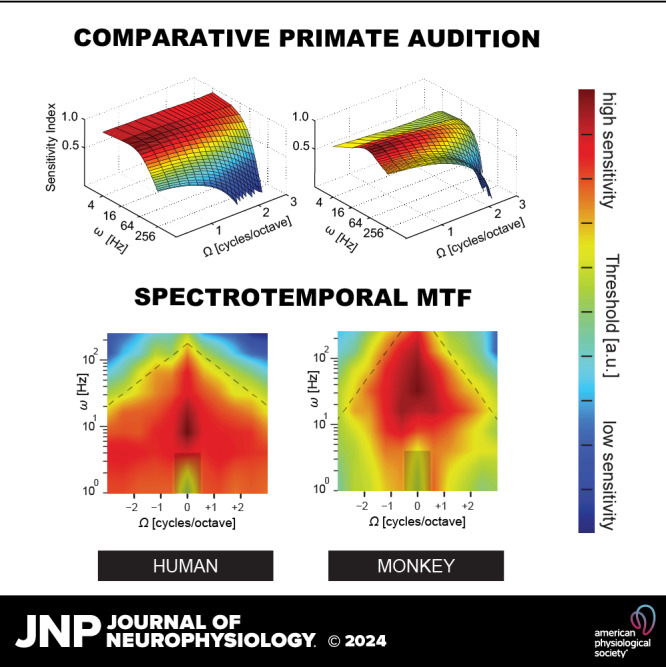

## INTRODUCTION

Biological sounds are characterized by statistical regularities in their dynamic spectral modulations, in which the frequency content changes over time. The ability to faithfully encode spectrotemporal (S-T) modulations is important not only for sound recognition but also for sound segregation in environmental noise, like listening to a conversation at a cocktail party (1–4). Similar problems arise when animals attempt to distinguish mating or echolocating calls from ambient noises (5, 6). Examples include species-specific communication signals in animals as diverse as mammals, birds, amphibians, reptiles, and insects (7–10). The auditory system faces the challenge to distinguish sounds based on their S-T modulation content. In particular, humans rely on the speed and direction of covarying S-T amplitude modulations to derive meaning from spoken words (4, 11).

**Figure 1.**
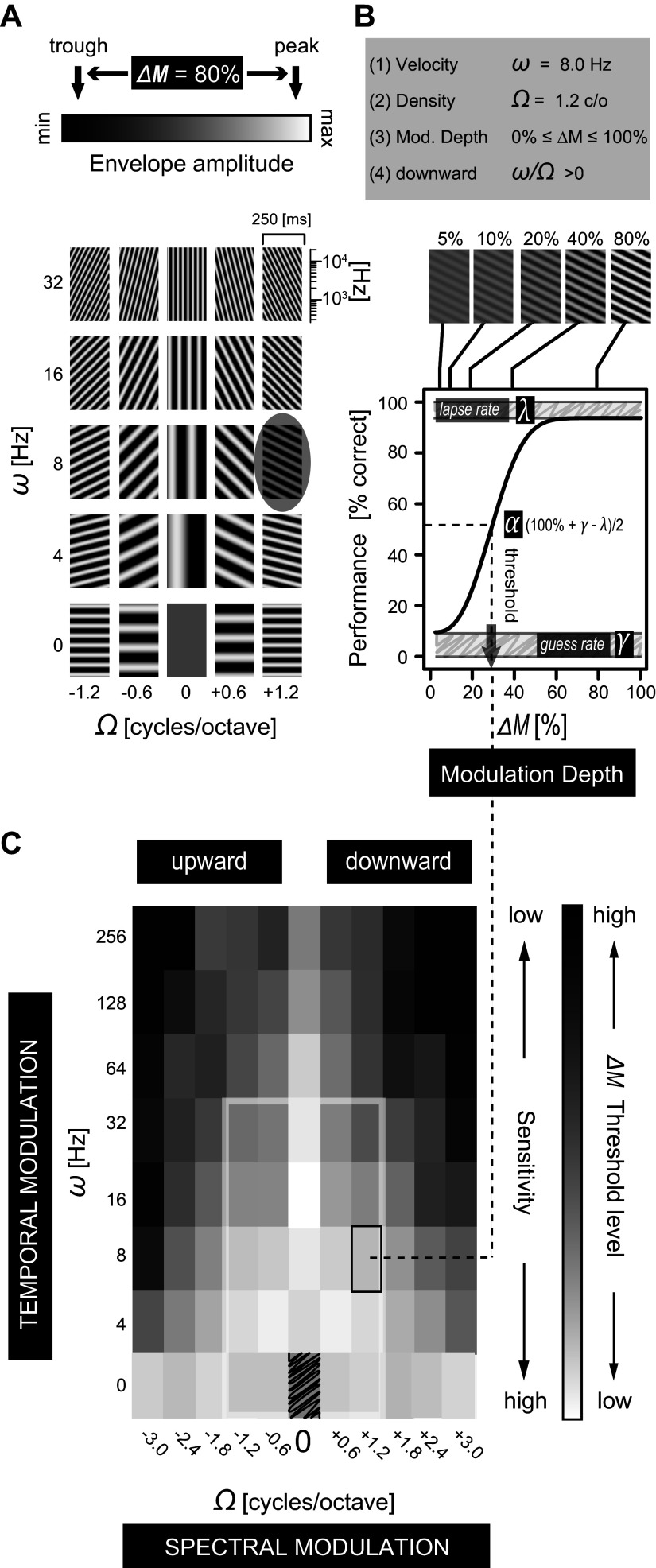
Dynamic rippled noise parameters and threshold S-T modulation transfer function (MTF) psychophysics. *A*: the 5 × 5 stimulus grid represents a subset of 25 ripple spectrograms with varying temporal (vertical axis) and spectral (horizontal axis) modulation rates. Three parameters define the amplitude envelope of each spectrogram: *1*) velocity ω (Hz): temporal modulation; *2*) density Ω (c/o): spectral modulation; *3*) modulation depth Δ*M* (%) (linear scale). The ω-to-Ω ratio specifies *4*) upward (<0) or downward (>0) direction of spectral motion. Ω = 0: pure temporal modulations; ω = 0: pure spectral modulations. The (0,0) stimulus has a flat spectrogram representing static noise. *B*: ripple onset detection performance as function of Δ*M*, for the encircled downward ripple in *A*. Detection threshold (*Eq. 5*) is defined at the half-point of the fitted (*Eq. 4*) psychometric curve (black line). The parameter α specifies the detection threshold response criterion, defining the function’s relative position along the *x*-axis. The guess rate (γ) and lapse rate (λ) specify the lower (close to 0%) and upper (close to 100%) bounds of the function, respectively. The here-estimated threshold level (arrow) equates to a modulation depth of ∼28%. *C*: the 87 ripples thus yield the threshold MTF as function of (Ω,ω), here shown for human *listener h1*. Gray scale represents Δ*M* threshold level, which is not defined for the (0,0) sound (hatched). Inner white rectangle circumscribes stimulus grid shown in *A.* The black outlined square at (1.2,8) represents the detection threshold as obtained from the fitted curve shown in *B*. See glossary for abbreviations.

Neurophysiological experiments in macaques implicate an ancient cortical system processing S-T modulations (12–16). The mechanisms by which monkeys process vocalizations could also extend to humans (17–22). With this comparative hypothesis in mind, we exposed humans and monkeys to a wide range of dynamic S-T ripples to characterize their S-T perceptual abilities (Fig. 1). Ripples (*Eqs. 1* and*2*) are naturalistic broadband signals with inseparable spectral and temporal modulations (Fig. 1*A*). They form a two-dimensional Fourier basis for sound, whereby any acoustic pattern can be composed by the superposition of a particular set of ripples (23, 24). Their importance in hearing research lies in the parametric assessment of auditory processing of complex sounds. Ripples have proven their audiological value as parametric nonspeech stimuli, responses to which are predictive for speech perception (25–27). Moreover, measuring auditory-evoked responses to ripples, at either perceptual or neurophysiological level, allows assessment of S-T (in)separability of, or within, the auditory system.

**Figure 2.**
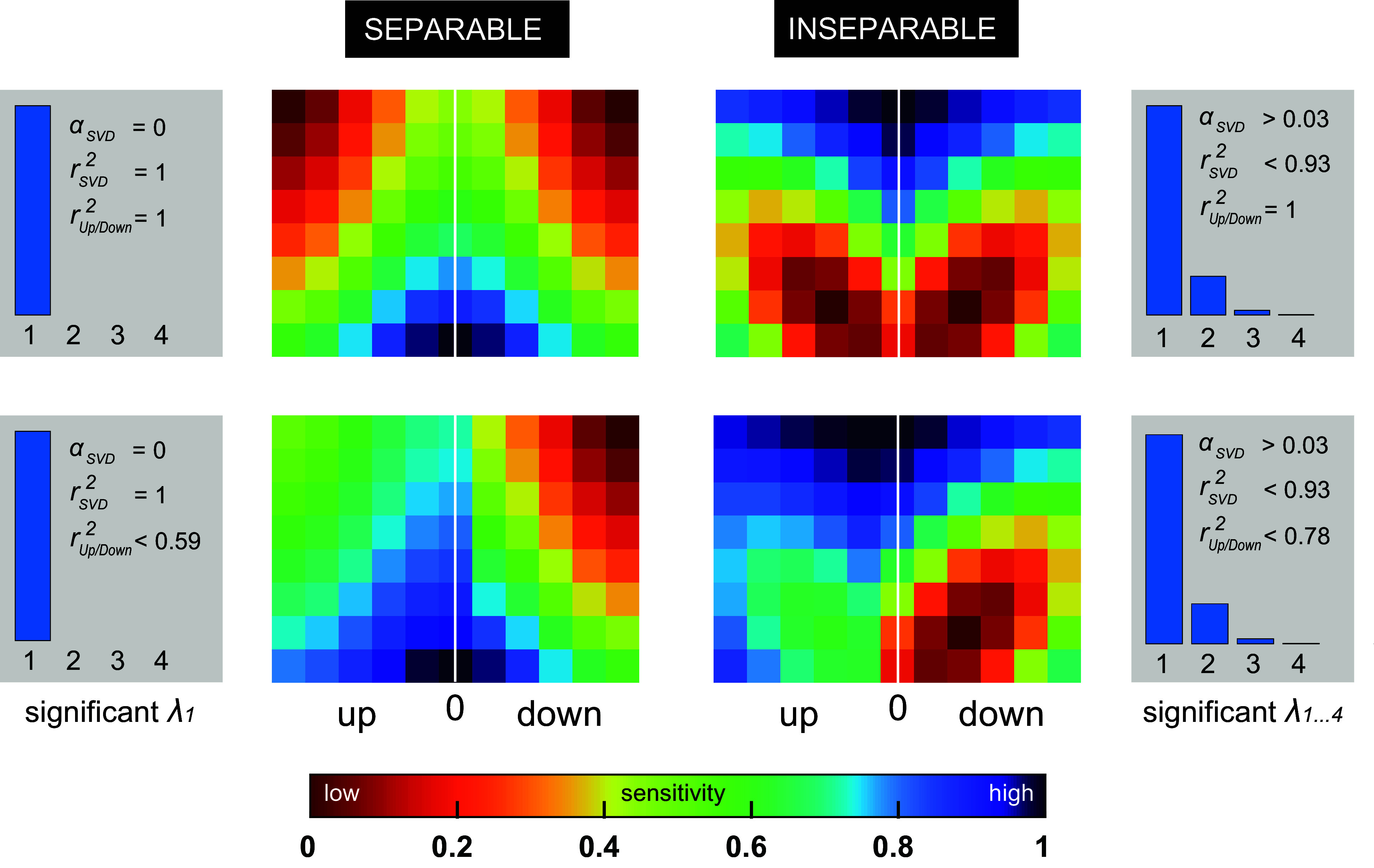
Separable vs. inseparable characterization of S-T hearing. Four theoretical sensitivity S-T MTF matrices (multicolored panels), equivalent to the gray-scaled one shown in Fig. 1*C*. Red colors specify low ripple sensitivity and blue colors high sensitivity. MTFs are categorized according to up/down symmetry, symmetric (*top*) vs. asymmetric (*bottom*) or extent of separability, separable (*left*) vs. inseparable (*right*) Note that the upper MTFs are mirror-symmetric around the zero-density (white vertical) axis and oriented orthogonal to the spectral modulation (horizontal) axis. The gray *insets* provide quantitative analysis of both the extent of inseparability and the up/down symmetry of the 4 displayed MTFs. Here, α_SVD_ reflects the degree of inseparability, with 0 corresponding to full separability across the entire S-T domain. The $r_{\mathrm{SVD}}^{2}$ statistic reflects the proportion of variance accounted for when assuming separability. Fully separable means that only the 1st eigenvalue, λ_1_, is significant (*Eq. 7*). Inseparable means that >1 eigenvalue is significant. The number of blue bars in each gray *inset* represents the number of significant eigenvalues needed to fully reconstruct their respective MTFs. The length of each bar is a measure of magnitude [arbitrary units (a.u.)]. The $r_{\mathrm{up/down}}^{2}$ statistic reflects perfect symmetry when equaling unity, and the gain *g* of the relation *M*(Ω *<* 0) = *g*·*M*(Ω *>* 0), equals 1. Note that the highly asymmetric up/down MTFs (*bottom*) display a sensitivity biased toward downward-moving ripple sounds, with a gain of 2.5. See glossary for abbreviations.

Separable or, alternatively, inseparable S-T sensitivity can be determined through singular value decomposition (SVD) analysis of the two-dimensional (2-D) S-T modulation transfer function (MTF; Fig. 1*C*) encompassing the product of a time-dependent [temporal modulation: velocity ω (in Hz)] and a frequency-dependent [spectral modulation: density Ω (cycles/octave, or c/o)] transfer function (*Eq. 7*). Separable S-T sensitivity is characterized by the inseparability index α_SVD_ (*Eq. 8*) equaling zero and the SVD MTF correlation coefficient $r_{\mathrm{SVD}}^{2}$ equaling unity, when separability is complete (see Fig. 2, *left*, for explanation). In this case, spectral and temporal modulations are processed independently. In contrast, inseparable S-T sensitivity is characterized by α_SVD_ > 0 and $r_{\mathrm{SVD}}^{2}$ < 1 (Fig. 2, *right*), highlighting that spectral and temporal modulations are processed dependently to some extent. Finally, S-T sensitivity can be biased to a particular ripple movement direction, upward versus downward S-T modulations, in which case the MTF sensitivity distribution is asymmetric along the horizontal dimension and could give rise to a $r_{\mathrm{up/down}}^{2}$ < 1 (Fig. 2, *bottom*).

Quantitative analysis of S-T receptive fields (STRFs) of auditory neurons has demonstrated an increased proportion of neurons with inseparable STRFs ranging from midbrain inferior colliculus (IC) to primary auditory cortex (13, 23, 24, 28–37). Although it is evident that separable and inseparable S-T encodings are manifest at different processing stages within the auditory pathway, it is not straightforward to predict what happens at the perceptual level. Psychophysical measurements in humans (38), assigning detection thresholds to a wide range of dynamic ripples, are consistent with an up/down symmetric, separable processing model (Fig. 2, *top left*). In this special case, the perceptual MTF is mirror symmetric around the zero-density axis and oriented orthogonal to the spectral modulation axis.

Given S-T separability of human hearing at threshold (38, 39), it is perhaps surprising to learn that the region with highest sensitivity is not optimized to the S-T modulations that dominate speech (4, 11, 12). Likewise, zebra finches show ripple detection thresholds (40) that do not correspond to the dominant modulation spectra of their own vocalization calls (37, 40). This is unexpected, since the forebrain of songbirds appears to be specialized for processing vocalizations (41).

Two hypotheses could explain these apparent discrepancies. First, preferential sensitivity to conspecific vocalizations may not be evident at the modulation detection threshold, as intelligible vocalizations are typically produced well above threshold (42). If so, suprathreshold MTFs could mirror the asymmetric nature of the S-T decompositions of, e.g., English speech (“intelligible”), wherein the strongest modulations are downward moving (38). Suprathreshold S-T hearing is then asymmetric, resembling the S-T sensitivity pattern of the bottom panels in Fig. 2 Alternatively, the processing of S-T modulations may be based on information efficiency principles (43, 44) instead of neuro-ethological ones (40). In this case, increased S-T sensitivity for vocalizations over other classes of biological sounds and perceptual levels is no longer expected and may give rise to a separable and symmetric MTF, also for suprathreshold sounds (Fig. 2, *top left*). To dissociate between preferential and nonpreferential sensitivity to naturalistic S-T modulations, and to enable a direct comparison between species, we studied five humans and five monkeys responding to a wide range of ripples under identical psychophysical conditions, and we determined their S-T sensitivities at threshold and suprathreshold levels.

Our results demonstrate that monkeys and humans share a largely unbiased up/down perceptual strategy, based on separable sensitivities to spectral and temporal amplitude modulations, when processing inseparable sounds. However, our analysis also indicated a small but significant contribution of inseparability to the S-T sensitivity of both species. To conclude, we also demonstrate, by means of SVD analysis of the known visual spatiotemporal CSF (45), that human vision is predominantly governed by inseparable processing of naturalistic stimuli.

## MATERIALS AND METHODS

### Ethics Statement

Our tests were purely behavioral and involved no distress or discomfort to our human volunteers or our monkeys. All experimental procedures complied with the European Communities Council Directive of September 22, 2010 (2010/63/EU). The local ethics committee for the use of laboratory animals (DEC) of the Radboud University reviewed and approved all experimental protocols. To ensure the animals’ health and welfare, their general appearance was monitored daily and recorded in a welfare diary, along with their daily food and fluid intake.

Human psychophysics on five healthy volunteers was performed after they had been informed about the behavioral procedures and their written informed consent was taken. Protocols were approved by the local ethics committee of the Faculty of Social Sciences of the Radboud University (ECSW 2016-2208-41).

As previously described (46, 47), the monkeys were pair-housed to stimulate normal social behavior. About 24 h before the start of a test session, water intake was limited to 20 mL/kg. The monkey earned a small water reward of 0.2 mL per successful test trial. After a test session, if needed, water was supplemented to the required minimum of 20 mL/kg and, in addition, the animal received pieces of fruit. On weekends, the animals’ fluid intake was increased to 400 mL daily.

To monitor the animal’s health status, body weight and water and food intake were recorded daily. Expert veterinarian assistance was available on site. Quarterly recording of hematocrit values ensured that the animal’s kidney function remained within the normal physiological range. When signs of discomfort or illness were observed, experiments were stopped and the animal was treated until it recovered. Our procedures follow the water-restriction protocol of the Animal Use and Care Administrative Advisory Committee of the University of California at Davis (UC Davis, AUCAAC, 2001).

### Participants, Animal Care, and Training

Five adult male rhesus monkeys (*Macaca mulatta*; weights 6.5–9.5 kg; *m1–m5*) and five adult humans (age between 23 and 43 yr; 1 female; 2 naive volunteers; *h1–h5*) participated in our experiments. Monkeys could move their head freely while seated in a custom-made primate chair within a sound-attenuated room. Each monkey learned to release a down-pressed bar upon the onset of an audible stimulus to receive a water reward. Psychophysical testing started when performance had stabilized and all monkeys could successfully complete a daily session with at least 1,500 trials.

### Pure-Tone Adaptive-Tracking Procedure and Stimulus Control

Tones (0.250, 0.375, 0.500, 0.750, 1.0, 1.5, 2, 3, 4, 6, 8, 12, 16, 24, and 32 kHz) were digitally synthesized and delivered online (260 kHz sampling rate) to a loudspeaker in the free field at the straight-ahead position (distance ∼80 cm), with Tucker Davis Technology’s hardware (TDT, Alachua, FL; RX6 Systems 3). Attenuation occurred through custom-built amplifiers. Loudspeaker output (Visaton GmbH; SC5.9) was sine-onset/cosine-offset ramped (5-ms rise/fall time) and defined by a flat frequency characteristic (to within 3 dB) from 0.1 up to 50 kHz after equalization (Behringer International GmbH, Willich, Germany; Ultra-Curve pro dsp8000). Sound intensity was calibrated by adjusting its root-mean-square (RMS) voltage with respect to a reference voltage [1 kHz at 80 dB sound pressure level (SPL)] and measured at the approximate position of the subject’s head with a calibrated Brüel and Kjær sound amplifier and microphone (B&K, Norcross, GA; BK2610/BK4134). Ambient background noise levels varied between 30 and 35 dB SPL. Reflections above 500 Hz were effectively attenuated by acoustic foam (Uxem, Lelystad, The Netherlands; Redux AX2250) covering the walls, floor, ceiling, and every large object present.

**Figure 3.**
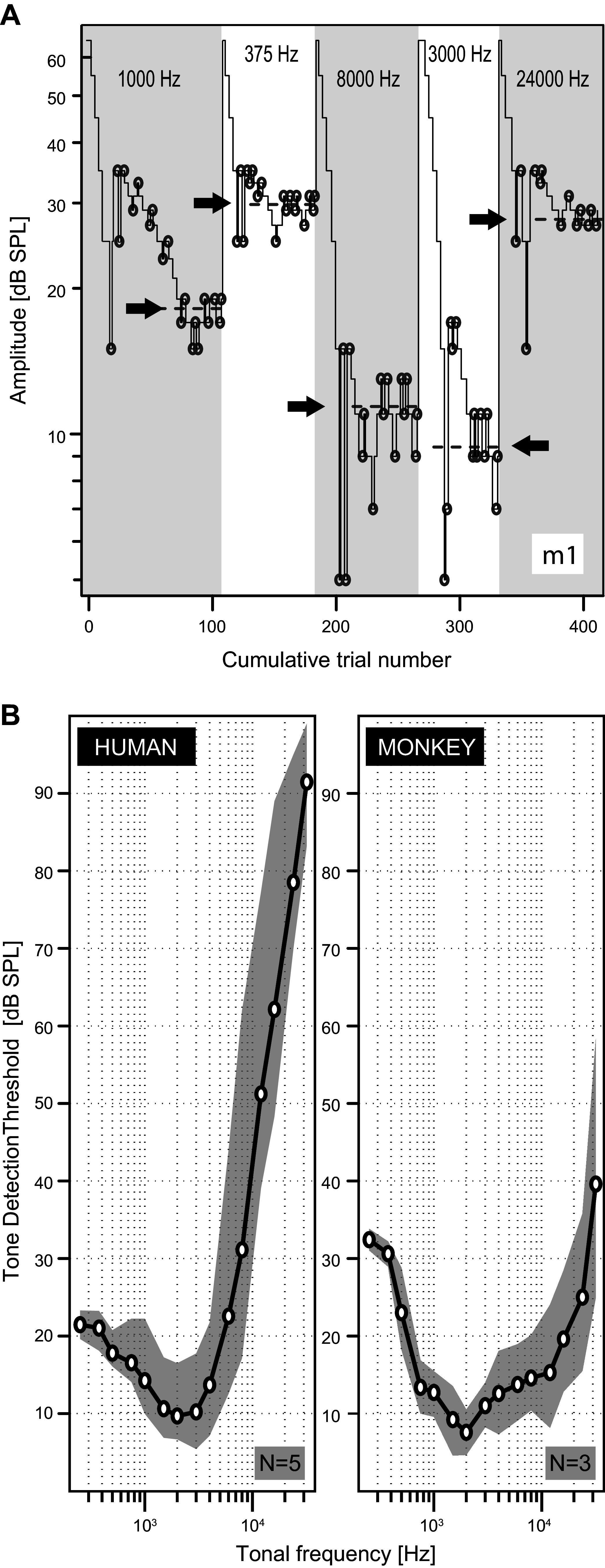
Tracking procedure and free-field audiograms. *A*: graphical representation of the adaptive-tracking procedure for *monkey m1*, as recorded on a single day. The thin black line shows 5 successive staircase runs produced by onset detection judgments to pure tones of 1,000, 375, 8,000, 3,000, and 24,000 Hz, respectively. Stimulus level was decreased after 3 consecutive hits and increased after 2 consecutive misses. A single run ended when at least 13 reversals (circles) occurred in the direction of the change in stimulus level. The average provided an estimate for the detection threshold as indicated by the arrows (dashed lines). This high level of stimulus control was observed in all monkeys tested (*m1–m3*). *B*: audiograms showing the hearing thresholds of human (*h1–h5*, *left*) and monkey (*m1–m3*, *right*) listeners, presented on a logarithmic scale. Gray areas indicate the 95% confidence intervals (95% CIs) as assessed by bias-corrected percentile bootstrap resampling on 100,000 evaluations. Our data concur with the known literature, showing that monkeys can hear sounds at frequencies that are much higher than humans can hear. Their hearing range extends to >20 kHz, whereas humans can only hear sounds up to ∼16 kHz.

Speaker-derived pure-tone thresholds were determined for all subjects, except for *monkeys m4* and *m5*, through a single-interval adaptive-tracking staircase procedure. Each staircase run commenced at 65 dB SPL and was adjusted according to the psychophysical transformed rule (48). That is, the intensity of a given tonal frequency was decreased by 10 dB after three consecutive hits, whereas it was increased by 10 dB after two consecutive misses. After four (monkeys) or two (humans) reversals the adaptive step size was reduced to 2 dB. Testing continued until at least 13 (monkeys) or 11 (humans) reversals had occurred for which the averaged intensity level was stable within 2 dB. Examples for five tones presented to *monkey m1* are shown in Fig. 3*A*.

The ability to perceive the onset of a pure tone was assessed by having listeners release a bar as soon as they heard the tone. We randomly varied the interstimulus time between 500 and 3,100 ms. All tones lasted 600 ms. Lapses in attention were monitored through catch trials, comprising a tone well above threshold. Catch trial tones had the same frequency as the staircase test stimulus with which they were randomly interleaved. Monkeys received ≈35% and humans ≈5% catch trials. Through this high percentage of catch trials the probability of being rewarded was 0.6, which ensured the monkey’s motivation to perform at high level. Staircase runs with lapse rates above 10% were discarded. Hearing thresholds were measured daily, with the 15 tonal frequencies presented in a random order to avoid bias. The final threshold estimates combined the data from 6 × 15 (per monkey: *m1–m3*) or 2 × 15 (per human: *h1–h5*) staircase runs that did not deviate >10% from the mean value (Fig. 3*B*).

**Figure 4.**
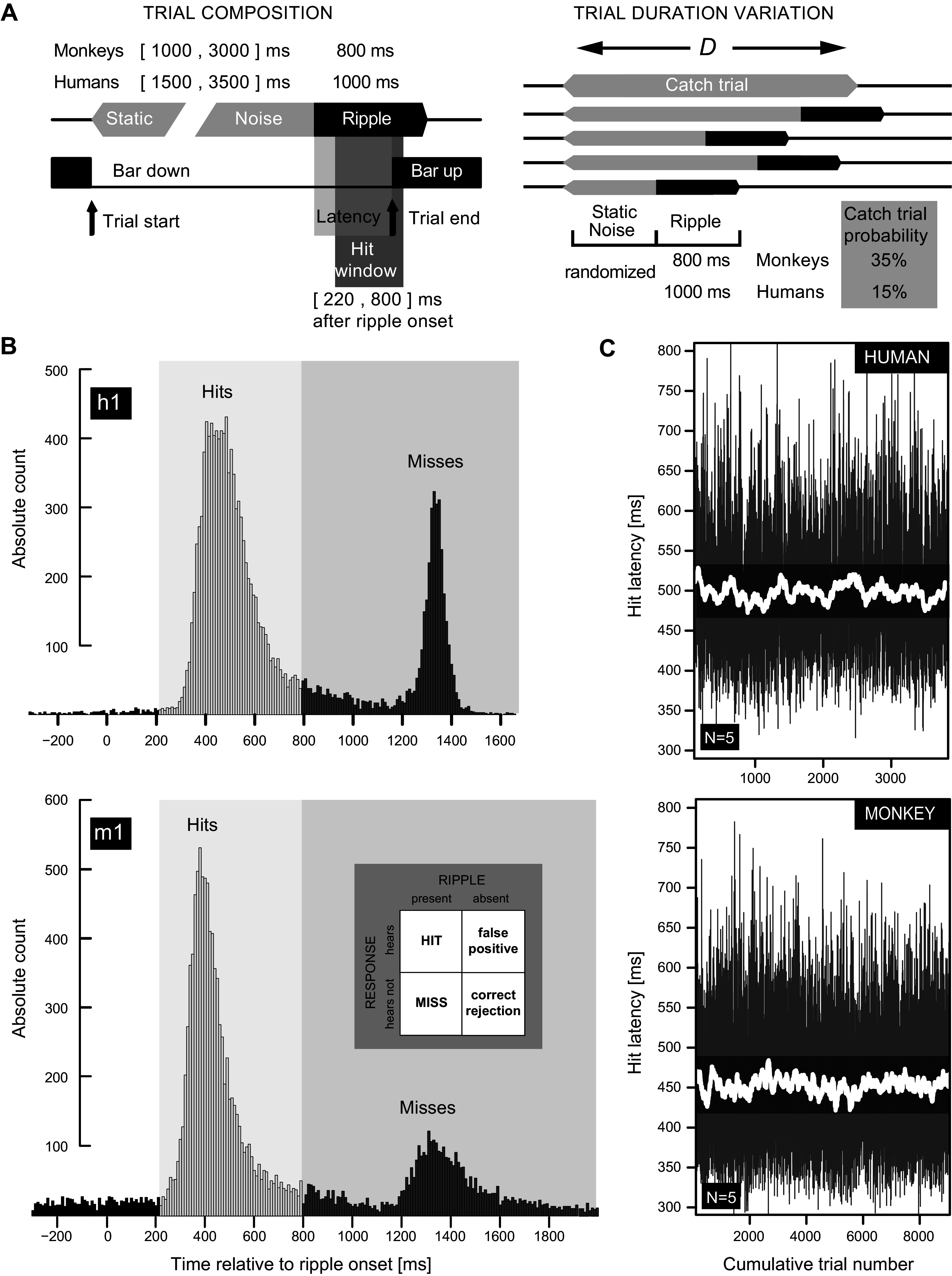
Behavioral paradigm and hit latency analysis. *A*: definition of hits and misses, trial duration (*D*), and catch trials. A trial started by holding down a response bar and terminated when the bar was released (vertical arrows) upon ripple onset detection. Static noise duration was randomized. Ripple duration remained constant. Responses between 220 and 800 ms after ripple onset were defined as hits (Hit window). When subjects failed to detect the modulation (latency > 800 ms), the response was counted as a miss. Early bar release trials (latency < 220 ms) were discarded. Note that catch trials comprised static noise only. *B*: distribution of reaction times for *human h1* (*top*) and *monkey m1* (*bottom*). The first peak corresponds to hits. The latencies around the second peak are due to misses. Data were collected over a period of 3–6 mo. The *inset* (dark gray, *bottom*) shows a 2 × 2 contingency table summarizing the 4 possible stimulus-response outcomes. Note that the probability of a hit response on catch trials when the ripple onset cannot be detected (false positive) is a measure of a listeners’ guess rate defining the lower bound (γ) of the fitted psychometric curve (see Fig. 1*B*). *C*: hit reaction times as function of cumulative trial number, pooled across stimuli: human (*h1–h5*, *top*) vs. monkey (*m1–m5*, *bottom*) listeners. The data cover the last 4,200 (*top*) or 8,500 (*bottom*) recorded hits of each listener. Solid white lines represent the 30-trial running average as a function of cumulative trial number. Gray areas reveal the variability in the underlying mean latencies. Note absence of a learning effect since the running averages do not decay over time.

Finally, we performed Monte Carlo simulations to emulate the performance of an ideal observer, not limited by internal noise constraints (49), responding to a single-interval hold-release task version of our three-down/two-up transformed rule. These simulations are needed because our single-interval task, equivalent to the one shown in Fig. 4*A*, essentially equates to a simple nonforced yes-no task for which there is no expected probability of the stimulus appearing at a given point in time, as opposed to a two-interval forced choice task where the probability of the stimulus presence equals 0.5 (48). For 100,000 simulations each containing up to 100 adaptive steps, the mean proportions of correct responses were found to range from 55% to 65%, with an average of 60%.

### Ripple Sound Design and Parameterization

Our test sequences with the ripple stimuli comprised a flat broadband noise of duration *D* followed by a S-T modulated broadband complex. Each ripple, *S*(*t*), included 126 simultaneously presented tones equally spaced, 20 per octave, along the logarithmic frequency scale, ranging from *f*_0_ = 250 Hz to *f*_126_ = 19 kHz (spanning 6.25 octaves):

(*1*) $$\;S(t)=\{\begin{array}{ccc} \;\sum_{n=1}^{126}R\left(t,x\right)\cdot \sin\;(2\pi \;\cdot f_{n}\cdot \;t\;+\;\;\varphi_{n}) & \text{for} & -\pi <\varphi_{n}<+\pi \\ \text{with}\;\;f_{n}\;=\;f_{0}\cdot \;2^{\frac{\left(n\;-1\right)}{20}} & \text{for} & 1\leq n\leq 126 \end{array}$$

Apart from the *f*_0_ component, which had its phase fixed at maximum amplitude (φ_0_ = π/2), tonal phase φ*_n_* was randomized between −π and +π. Noise amplitude was modulated by a single sinusoidal envelope, *R*(*t,x*):

(*2*) $$\;R(t,x)=\{\begin{array}{ccc} 1 & \text{for} & 0\leq t<D\\ 1+\Delta M\cdot \;\sin\;\left[2\pi (\omega \cdot t+\Omega \cdot x)\right]\; & \text{for} & 0\leq t<D\\ \text{with}\;\;x=\frac{n-1}{20} & \text{for} & 1\leq n\leq 126\;\;\; \end{array}\;\;\;\;\;\;\;\;\;\;\;\;\;\;\;\;$$

Here, *t* is time (seconds); *x* is the position on the frequency axis in octaves above *f*_0_; ω is the temporal modulation rate, called ripple velocity (Hz); Ω is the spectral modulation rate, called ripple density [cycles/octave (c/o)]; Δ*M* is the envelope amplitude modulation depth of the ripple on a linear scale from 0 up to 100%; and *D* defines the duration of the static noise (ω and Ω are set to 0) at the onset of the stimulus sequence. In the modulated second part (*t > D*), the sign of the ω-to-Ω ratio sets the upward (<0) or downward (>0) direction with which the amplitude envelope sweeps the S-T domain. As illustrated in Fig. 1*A*, pure temporal amplitude modulations, Ω = 0, give rise to vertically oriented modulations, called amplitude-modulated (AM) noises. Pure spectral modulations, ω = 0, give rise to horizontally oriented modulations, called static ripples (50). Sound level (RMS) was fixed at 56 dB SPL for both the static noise and the ripple. As detailed in Fig. 4*A*, *D* was varied from 1.0 to 3.0 s in humans and from 1.5 to 3.5 s in monkeys. Modulation duration equaled 0.8 s in humans or 1.0 s in monkeys. The longer duration for the monkeys was needed to ensure stimulus control at low modulation depth levels. Also, other studies have found that monkeys perform better when exposed to longer durations in temporally based auditory tasks (51).

All stimuli were selected from a matrix of 88 combinations of (*n* = 11) ripple densities Ω (−3.0, −2.4, −1.8, −1.2, −0.6, 0, +0.6, +1.2, +1.8, +2.4, and +3.0 c/o) and (*n* = 8) ripple velocities ω (0, 4, 8, 16, 32, 64, 128, and 256 Hz). A subset of this matrix is shown in Fig. 1*A*. Up to 11 Δ*M* levels were used (0, 5%, 7.5%, 10%, 15%, 20%, 30%, 40%, 50%, 70%, and 100%). As the catch trial stimulus (Ω,ω) = (0,0) was not modulated, subjects heard 10 × 87 + 1 = 871 different audible S-T sounds.

**Figure 5.**
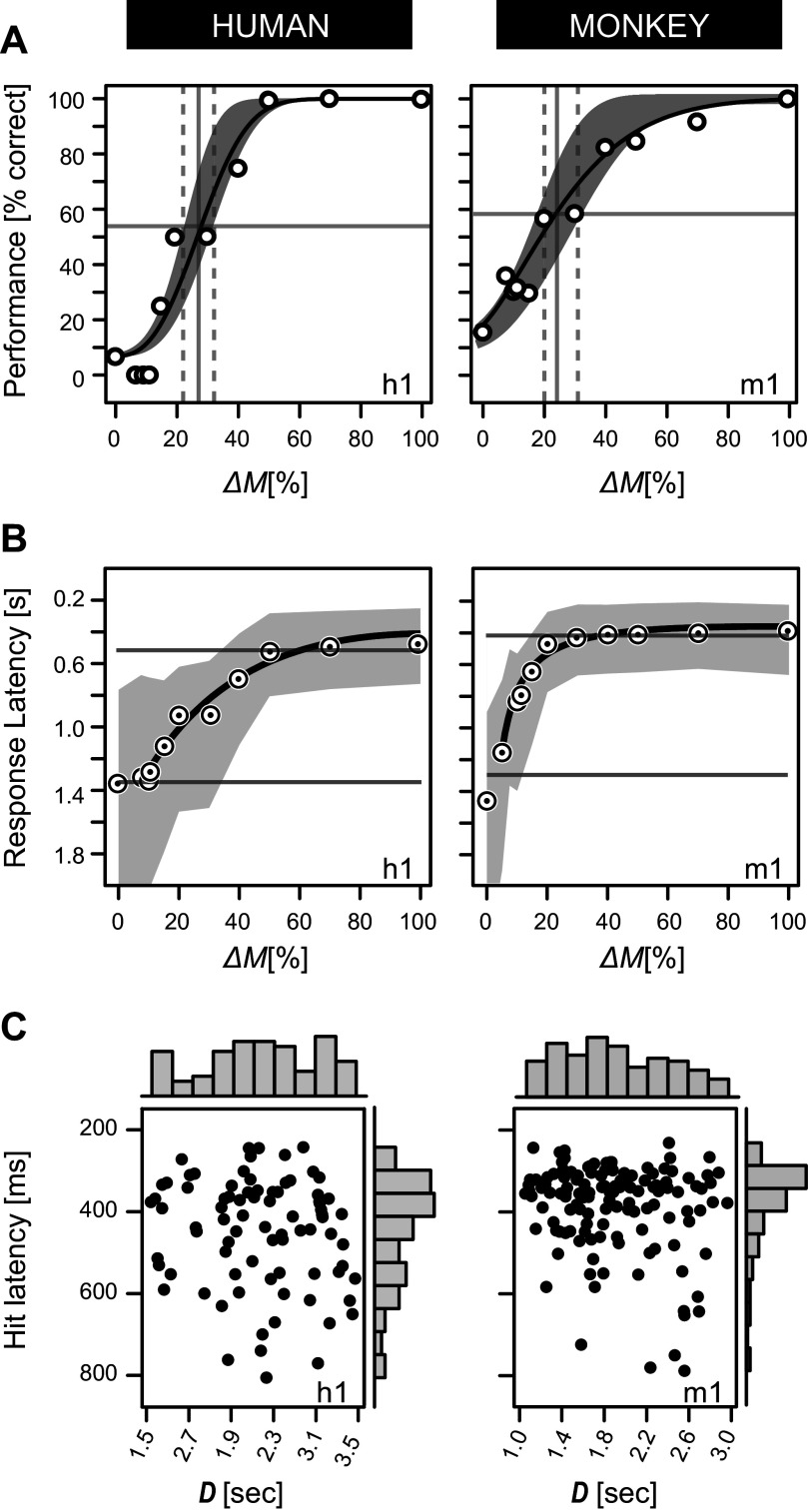
Ripple detection threshold and latency analysis. *A*: fitted psychometric curves (*Eq. 4*) of *listeners h1* and *m1* for dynamic ripple Ω (−3.0 c/o) and ω (32 Hz). Vertical dashed lines define the 95% CI of the detection thresholds. The gray areas comprise 10,000 evaluations of the expected performance function, obtained through Monte Carlo resampling. Trial sample sizes: *n_h1_* > 11 × 12; *n_m1_* > 11 × 18. *B*: ripple onset detection latency as a function of Δ*M* to the same ripple. The black lines were based on a shape-preserving interpolation algorithm for illustrative purposes only. Gray areas define the 95% CIs, as assessed by bias-corrected percentile bootstrap resampling (100,000 samples). The *top* and *bottom* horizontal gray lines mark peaks of hits and misses of the reaction time distributions in Fig. 4*B*. *C*: static noise duration (*D*) and ripple onset reaction time (latency) of *human h1* and *monkey m1* are unrelated. Histograms indicate near-uniform distributions of *D* (vertical gray bars) and nonuniform distributions of latency (horizontal gray bars). See glossary for abbreviations.

Sound synthesis, digitalization, deliverance (50 kHz sampling rate), and acoustic conditions were identical to those described for the tone audiogram above, except that each stimulus sequence was stored offline as a waveform audio file before the experiment proper. Sound level was 56 dB SPL. The following methodological requirements were met: First, each subject received a unique set of *n* × 871 (*D*, Δ*M*, ω, Ω) combinations distributed evenly over the recording sessions. Second, *D* was uniformly distributed over the (Δ*M*, ω, Ω) combinations (Fig. 5*C*). Third, the order in which the test sequences were presented was unique for each subject. Fourth, sound intensity and total power of the flat broadband noise equaled those of the ripple.

Finally, we calculated the normalized 4th moment, *M*4, of our ripple stimuli as defined by

(*3*) $$M4=\frac{\frac{1}{T}\int_{0}^{T}S^{4}(t)dt}{{\left[\frac{1}{T}\int_{0}^{T}S^{2}(t)dt\right]}^{2}}$$

where *S*(*t*) is the time-domain representation (*Eq. 1*) and *T* is the duration. *M*4 is of behavioral relevance because it provides a measure of instantaneous amplitude fluctuations to which humans are known to be quite sensitive (52).

**Figure 6.**
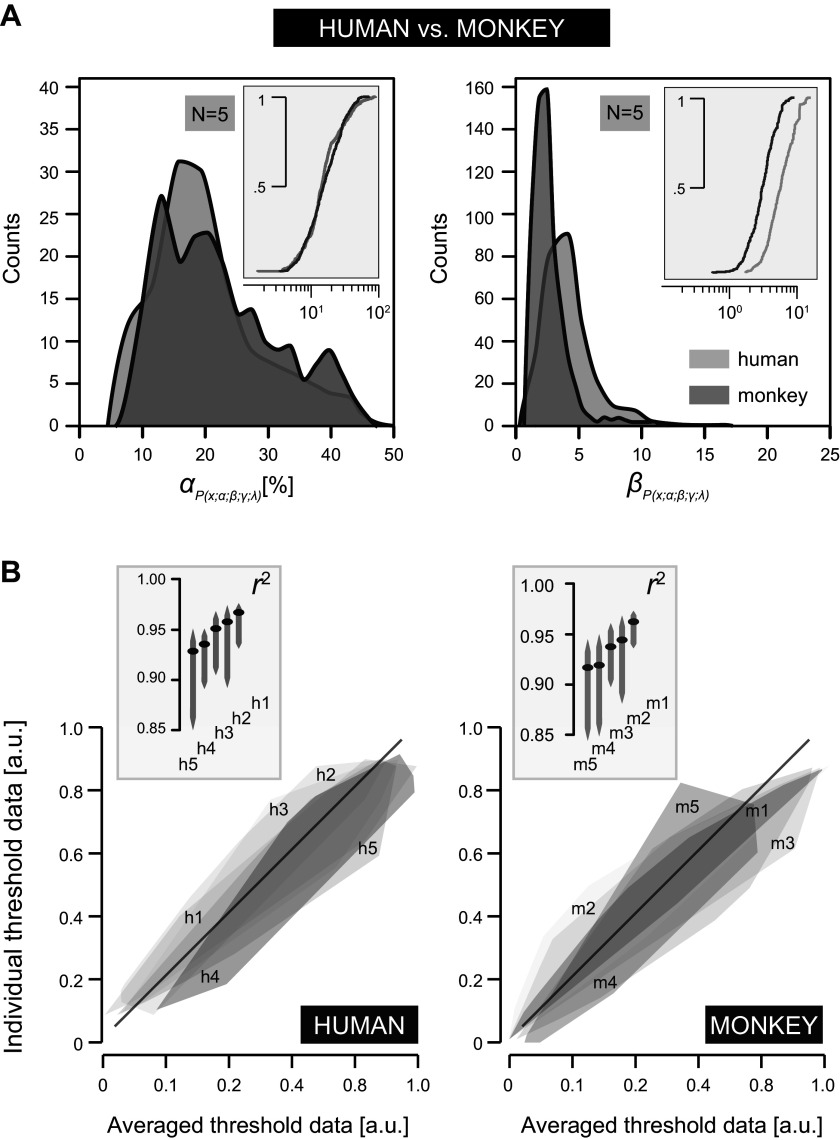
Psychometric threshold and slope value distributions. *A*: distribution plots of the fitted psychometric function (*Eq. 4*) parameters α (threshold, *left*) and β (slope, *right*). Data are pooled across human (*h1–h5*; light shading) or monkey (*m1–m5*; dark shading) listeners, representing 2 × 5 × 87 data points per plot. *Insets* represent log-scaled cumulative distributions of the associated probability density functions. Note that the α distributions fully overlap; the β distributions have a narrow width (<5), whereby monkeys yield systematically lower slopes. *B*: comparison between α values of individual subjects and the pooled averaged thresholds (*humans*, *left*; monkeys, *right*). Gray shaded areas represent the convex hull enclosing all 87 data points as obtained from each listener. Thresholds are scaled onto the [0,1] range (*Eq. 6*). The diagonal (black) corresponds to the identity line. *Insets* represent the 95% CIs (gray lines) of the coefficients of determination (black dots) corresponding to the data of each listener shown below. None of the 95% CIs has a value below 0.8, highlighting a close relationship between the individual and the averaged datasets. a.u., Arbitrary units. See glossary for additional abbreviations.

The averaged log_10_(*M*4) value pooled over 11 Δ*M* values {mean [95% confidence interval (CI)]} of unmodulated noise, dynamic ripples and static ripples were (2.99 [2.98–2.99]), (2.98 [2.98–2.99]), and (4.2 [3.96–4.55]), respectively. Thus, static ripples stand out from the dynamic ripples in the sense that they could in principle be discriminated on the basis of their higher *M*4. However, for Δ*M* ≤ 55% (still well above threshold, see Fig. 6*A*), the *M*4 of static ripples did not deviate significantly from those obtained from flat noise or dynamic ripples; thus listeners could not use instantaneous fluctuations as a potential cue.

### Ripple Detection Paradigm, Stimulus Control Monitoring, and Number of Trials

We assessed perceptual performance by requiring listeners to release a response bar upon detection of an audible change in an otherwise flat broadband noise. A trial started by pushing down a response bar and terminated when the bar was released upon ripple onset detection. Responses between 220 and 800 ms after ripple onset were defined as hits. When subjects failed to detect the modulation (latency > 800 ms), the response was counted as a miss. Early bar release trials (latency < 220 ms) were discarded (Fig. 4*A*).

We used the method of constant stimuli to measure the S-T modulation detection performance to all 88 (Ω, ω) combinations as a function of 11 stimulus levels, Δ*M*. Daily, stimulus levels were presented in a randomly intermixed sequence from a predefined subset of randomly selected (Ω, ω) combinations to form a single recording session. Daily recording sessions contained ≈1,600 responses for monkeys, as opposed to ≈600 for humans.

**Figure A1.**
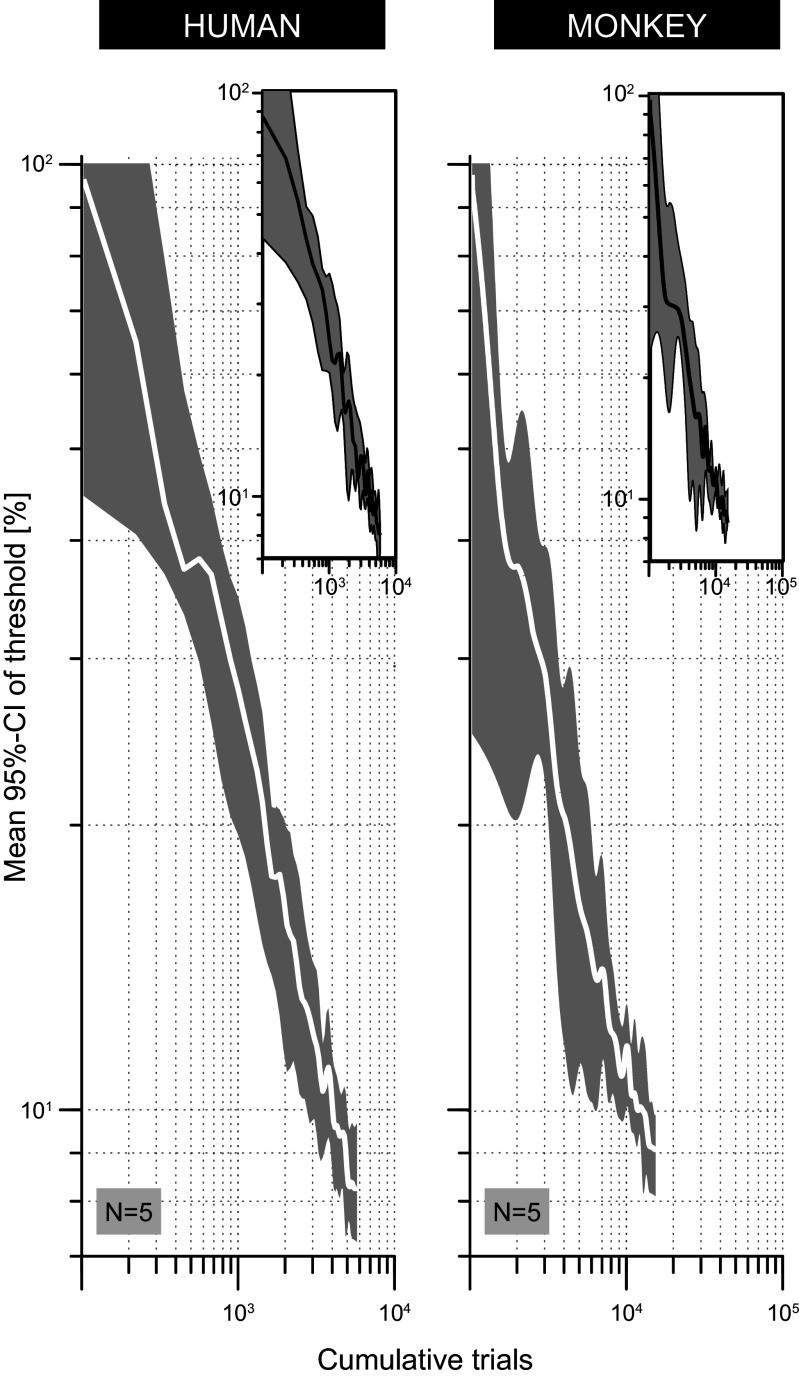
Solid lines represent the mean 95% CIs of 5 × 87 ripple threshold values as a function of the number of cumulative trials for human (*left*, *h1–h5*) and monkey (*right*, *m1–m5*) listeners. The cumulative trial number can be regarded as a measure of “practice time.” Gray areas define the 95% CI, as assessed by bias-corrected percentile bootstrap resampling (100,000 samples). Data were smoothed for illustrative purposes only. *Insets* show the same data used to compute the solid white lines, but now the chronological order with which the data were collected was reversed. Note that in this case (solid black lines) the same number of trials was required to converge to the same confidence levels. These graphs show that performance was stable over time and that the decline of CIs with time resulted from the accumulation of data.

To monitor stimulus control in monkeys, pure static noise catch trials, (Ω,ω) = (0,0), presented at a probability *P* ≈ 0.35, were randomly interleaved with the test sequence trials. Human listeners received catch trials at *P* ≈ 0.15. Catch trial performance, i.e., a measure of the listeners’ guess rate, ranged from 16% up to 35% in monkeys. For humans, this range was 1% to 12%. We observed that guess rates were roughly constant for both species and within the expected range known from the literature. In addition, we found that procedural and perceptual learning did not have a long-lasting effect on performance in both humans and monkeys. This is shown in Fig. A1 in *Robustness of Detection Threshold Estimation* in the appendix.

In total, each of the 968 ripple stimulus parameter combinations, Δ*M*, ω, and Ω, was repeated at least 16 times for monkeys and 8 times for humans. Measurements were terminated when the 95% CI of all 88 (Ω,ω) thresholds (*Eq. 5*) was <10%.

Overall, monkeys performed in >19,000 trials, which were spread out over 20–30 daily recording sessions. Humans, on the other hand, performed in ∼8,000 trials or more, which were spread out over 13–16 recording sessions. The total number of responses required to obtain reliable threshold estimates (95% CI < 10%) was higher for the monkeys (*m1–m5*: 19,721–23,291) than for the human listeners (*h1–h5*: 8,481–8,811).

### Psychometric Function Parameterization and Fitting

Our single-interval psychometric functions, *P*(*x*;α;β;γ;λ), were parameterized as cumulative Weibull distribution functions *F*(*x*;α;β):

(*4*) $$P(x;\alpha ;\beta ;\gamma ;\lambda )=\{\begin{array}{c} \gamma +(1-\gamma -\lambda )\cdot F(x;\alpha ;\beta )\\ \text{with}\;F(x;\alpha ;\beta )=1-e^{-(x/\alpha )\beta }\;\text{for}\;0\leq x\;\leq \;100 \end{array}$$

Here *x* is the dependent variable Δ*M*; γ is the *guess rate* (false positives), representing the fraction of trials where listeners released the bar at random but within the hit window time interval (as defined in Fig. 4*A*); λ is the *lapse rate* (misses), calculated from the difference between 100% correct and the actual performance at near-maximum Δ*M* values. Thus, γ and λ define the lower (close to 0%) and upper (close to 100%) bound of the psychometric function, respectively (as indicated graphically in Fig. 1*B*). The parameter α specifies the threshold response criterion, defining the function’s relative position along the *x*-axis, and β specifies the slope (lateral spread) of the cumulative Weibull distribution function. The detection threshold was defined as the Δ*M* value of *x* for which responses fell on the midpoint between the lower and upper bound of the fitted psychometric curve (Fig. 1*B*):

(*5*) P(x;α;β;γ;λ)=γ+(1−γ−λ)/2

The four parameters, α, β, γ, and λ, were treated as free parameters. As Bayesian constraining prior functions (53) we chose beta distributions for λ and γ, normal distributions for α, and log-normal distributions for β. The log-likelihood ratio, based on 10,000 Monte Carlo simulations, allowed verification of the goodness of fit: two-sided $\chi_{\mathrm{derivative}}^{2}$ > 20, *P* < 0.003. That is, the likelihood of finding a deviance greater than 20, given 11 stimulus levels and 4 free parameters (i.e., number of degrees of freedom equals 7), by chance alone for all of the 880 fitted psychometric functions (pooled across all 10 subjects) was <0.3% (54, 55). Cross-validation analysis by means of Bayesian inference and model-free estimation (55) on 10% (randomly selected) of the performance data collected did not produce thresholds and slopes with significantly different 95% CIs. In other words, the estimated thresholds as derived from the raw performance data did not depend on the statistical method used.

Blocks of trials in which monkey listeners did not reach 100% detection level at easily detectable ripple modulation levels, i.e., lapses in attention judged on misses (lapse rate) to Δ*M >* 80%, were discarded (56, 57). This was determined through visual inspection of the upper bound of the fitted psychometric curves (*Eq. 4*). About 40–20% of the daily recorded monkey responses were discarded. This level of inattention is not uncommon for trained macaque monkeys (51, 58, 59).

### Threshold S-T MTF Construction

From the ripple detection performance data of each listener, as obtained for each of the 87 (Ω, ω) detectable combinations, we fitted a psychometric function (*Eq. 4*) using the constrained maximum-likelihood algorithm (54). Ultimately, all 87 functions were used to construct the threshold S-T MTF matrix *M*(Ω,ω), as shown graphically in Fig. 1. The threshold value for the stimulus at (0,0), for which the threshold cannot be determined because it is inherently indistinguishable, was determined through interpolation.

### Suprathreshold S-T MTF Construction

We obtained suprathreshold MTFs by constructing iso-Δ*M* MTFs from the complete database of psychometric functions. That is, instead of using the performance scale, the threshold response criterion (*Eq. 5*) used was the stimulus scale Δ*M* as the independent measure for constructing suprathreshold MTFs.

### MTF Normalization

To enable a visual and direct quantitative comparison across subject and between species (Fig. 8*A*, Fig. 12*A*, Fig. 13, and Fig. 14), we normalized all values of MTF matrix *M*(Ω,ω):

(*6*) $$\begin{array}{ccc} M_{\mathrm{norm}}\left(\Omega ,\omega \right) & = & \frac{M(\Omega ,\omega )-\min\;[M(\Omega ,\omega )]}{\max\;[M(\Omega ,\omega )]-\min\;[M(\Omega ,\omega )]} \end{array}$$

with max[*M*(Ω,ω)] and min[*M*(Ω,ω)] representing the highest and lowest values of the MTF of each listener. In this way, each value was scaled onto a [0,1] range.

### Inseparability Index α_SVD_

The degree of separability was quantified for each *M*(Ω,ω) through singular value decomposition (SVD) (30, 36), expressing *M*(Ω,ω) as the product of three matrices:

(*7*) $$M\left(\Omega ,\omega \right)=\text{G}\left(\omega \right)\cdot K\left(\lambda_{i}\right)\cdot H\left(\Omega \right)$$

**G**(ω) and **H**(Ω) are orthogonal matrices, and **K**(λ*_i_*) is the singular matrix with the dimensionless eigenvalues λ*_i_* on its diagonal and zeros elsewhere. If the singular matrix has only one significant eigenvalue, λ_1_ > 0 and λ*_i > _*_1_ = 0, then *M*(Ω,ω) is fully explained by the product of two orthogonal vectors. These are the first singular vectors in *G*_Ω_(ω) and *H*_ω_(Ω), representing the temporal (TMTF) and the spectral (SMTF) modulation transfer functions, respectively. In other words, *M*(Ω,ω), is then said to be fully separable, when every row is a scaled version of every other row, and columns are scaled versions of each other, too.

The degree of separability was quantified with the inseparability index (21, 30, 36, 60):

(*8*) $$\alpha_{\mathrm{SVD}}=1-(\lambda_{1}^{2}/\sum_{i=1}^{n}\lambda_{i}^{2})$$

with summation over the number of tested velocity values, *n* = 8, as prescribed by matrix **M**(Ω,ω). Thus, α_SVD_ represents the proportion of the total power in *M*(Ω,ω) that is accounted for by its highest inseparable approximation. If α_SVD_ = 0, the power in the MTF is only determined by the first eigenvalue and thus separable. If α_SVD_ > 0, however, then *G*(ω) and *H*(Ω) may interact.

To test the statistical significance of α_SVD_ > 0, the α_SVD_ values computed from the actual *M*(Ω,ω) were plotted against those computed from randomly permuted versions of *M*(Ω,ω) (see, e.g., Fig. 9*A*). This was achieved by generating 100,000 bias-corrected percentile bootstrap (61) samples of α_SVD_ for both the actual and randomized data.

### Singular Value Decomposition S-T MTF: Coefficient of Determination $r_{\mathrm{SVD}}^{2}$

To quantify how a given α_SVD_ > 0 (Fig. 2, *right*: inseparable) relates to the degree with which the actual measured *M*(Ω,ω) can be reconstructed, we replaced the singular eigenvalue matrix **K**(λ*_i_*) of *Eq. 7* with only its first eigenvalue λ_1_. This yields the predicted or recovered MTF: *M*_rec_(Ω,ω), under the assumption of full separability. A quantified measure for the degree of separability, $r_{\mathrm{SVD}}^{2}$, was calculated by performing a Spearman’s rank correlation between each of the 88 elements of *M*_rec_(Ω,ω) and the measured *M*(Ω,ω). Thus, a fully separable MTF gives rise to an $r_{\mathrm{SVD}}^{2}$ that equals unity (Fig. 2, *left*: separable).

### Up/down Symmetry MTF: Coefficient of Determination $r_{\mathrm{up/down}}^{2}$

To quantify the degree of up/down symmetry, the MTF of a single listener was divided into a pair of half-matrices: one containing the thresholds (iso-Δ*M* scores) to upward *M*(Ω *<* 0, ω > 0)- and the other to downward *M*(Ω *>* 0, ω > 0)-moving ripples (see Fig. 2). The derived measure of squared correlation between upward and downward sensitivity, $r_{\mathrm{up/down}}^{2}$ = 1, reflects perfect symmetry when the gain *g* of the relationship *M*(Ω *<* 0) = *g* **×** *M*(Ω *>* 0) equals 1. Bootstrap analysis [100,000 bias-corrected percentile bootstrap samples (61)] of randomly permuted MTF matrices gave rise to $r_{\mathrm{up/down}}^{2}$ ≤ 0.8. Thus, $r_{\mathrm{up/down}}^{2}$ > 0.8 signifies a high degree of up/down symmetry that did not arise spontaneously.

### Mutual Information

We applied a mutual information analysis (see, e.g., Figs. 8*B*, 9*B*, and 12, *B* and *C*) to obtain a quantifiable measure of the geometric relationship between a pair of *M*(Ω,ω) matrices, like the ones shown in Fig. 8*A* (62).

Mutual information is defined as follows. For *X*, a discrete random variable with probability distribution *p*(*X*), the Shannon entropy (63, 64), in bits, is defined as

(*9*) $$H(X)=-\sum_{i=1}^{n}p_{i}\log\;p_{i}$$

Here *X* can take *n* discrete values *x_i_*,…, *x_n_* with corresponding probabilities *p_i_*,…, *p_n_*. Note that *H*(*X*) *≈ 0* when *p* ≈ 0 or *p* ≈ 1; otherwise *H*(*X*) *>* 0. The mutual information of *H*(*A*) and *H*(*B*) is then defined by

(*10*) I(A;B)=H(A)+H(B)−H(A,B)

where *H*(*A,B*) is the conditional entropy of *A* given *B*. If *A* and *B* are dependent variables, the total entropy is reduced. Mutual information is sensitive to both the size and the information content of the overlap between *A* and *B* (64). For further details, see *Mutual Information Computation* in the appendix.

### Probability Density Estimation

Nonparametric kernel density estimation methods (65) allow for optimal interpolation of finite data to construct a continuous representation (66). We used an adaptive MATLAB (The MathWorks, Inc., Natick, MA)-implemented algorithm, based on the smoothing properties of linear diffusion processes (67), to compute probability density functions.

### Random Permutation

Data were randomly permuted by means of the MATLAB *randperm* function. That is, the index numbers, row and/or column indices, of a given one-dimensional (1-D) data vector or two-dimensional (2-D) data matrix were reshuffled randomly.

### Kolmogorov–Smirnov Test

Two-sample Kolmogorov–Smirnov (KS; nonparametric) testing was performed to compare the empirical distribution functions of two continuous random variables (with sample size *n*) under the null hypothesis, *H*_0_, that both are from the same continuous distribution.

### Kendall’s Rank Correlation

Kendall’s rank correlation is a nonparametric test of independence. We calculated the Kendall’s tau correlation coefficient*, tau-b,* under the null hypothesis that there is no ordered relationship.

### Confidence Intervals

Confidence intervals (CIs) were estimated with a nonparametric, bias-corrected bootstrapping algorithm (61).

### Auditory Spectrotemporal MTF Modeling

Details on the computation of human and monkey spectrotemporal (S-T) MTF surfaces of Fig. 14 (audition: 2 plots at *top left*) are provided by *Eq. A2* in *Auditory Spectrotemporal MTF Modeling* in the appendix.

### Visual Spatiotemporal MTF Modeling

Details on the computation of the human space-time surface, *G*(α,ν) of Fig. 14 (vision: plot at *top right*) are provided by *Eq. A3* in *Visual Spatiotemporal MTF Modeling* in the appendix.

## RESULTS

### Pure-Tone Hearing Sensitivity

We determined free-field pure-tone audiograms to ensure that the listeners had normal hearing. Figure 3*A* shows an example of our psychophysical adaptive-tracking procedure with *monkey m1* for five different tones. Note the stable behavior around the different pure-tone thresholds, demonstrating that the animal was under full stimulus control. We obtained similar results for *monkeys m2* and *m3*.

Figure 3*B* shows the averaged hearing thresholds of all human listeners (*h1–h5*; Fig. 3*B*, *left*) and three monkeys (*m1–m3*; Fig. 3*B*, *right*) for all tested frequencies (0.125 ≤ *f* ≤ 32 kHz). Note that the monkeys’ hearing range extends to frequencies that are inaudible to humans (monkeys: threshold ∼40 dB at *f* = 32 kHz; humans: threshold > 90 dB). In conclusion, the mean range of our subjects’ audiograms corresponds well with normal hearing (68).

### Ripple Stimulus Paradigm and Latency Analysis

Listeners were trained (monkeys) or instructed (humans) to release a response bar upon detection of an audible change, i.e., ripple onset, in an otherwise static broadband noise of random duration. In total, we employed 87 combinations of spectral and temporal modulation rates (Fig. 1*C*), across 10 modulation depths, Δ*M* (Fig. 1*B*), plus a catch stimulus without S-T and amplitude modulation [(Ω,ω,Δ*M*) = (0,0,0)]. As such, each listener was exposed to a (pseudo)randomized sequence of 871 unique (Δ*M*,Ω,ω) ripple combinations, for which the timing of the ripple onset, dictated by duration *D* of the static noise, was unpredictable (horizontal gray bars, Fig. 4*A* and vertical gray bars, Fig. 5*C*). During testing, each ripple was repeated at least *n* = 16 (monkey) or *n* = 8 (human) times.

Figure 4*B* illustrates the complete latency distributions of *human h1* (8,811 responses) and *monkey m1* (19,721 responses). Both histograms reveal a clear bimodal distribution. The first peak, Hits, corresponds to correctly detected ripples. The averaged hit latency, median plus confidence interval [95% CI] in milliseconds, in our monkey (*m1–m5*) and human (*h1–h5*) listeners was 400 [366–412] ms and 443 [323**–**472] ms, respectively. These data correspond well to reaction times of sound-evoked hand/arm movements (69). The second peak, Misses, corresponds to ripples that listeners failed to hear.

The pooled latency data of Fig. 4*C* were selected for hits only and are displayed as a function of cumulative trial number across all recording sessions. Compared with our human listeners (*h1–h5*; Fig. 4*C*, *top*), the monkeys (*m1–m5*; Fig. 4*C*, *bottom*) were on average 43 ms faster in releasing the response bar upon ripple onset detection. However, neither the mean (white lines in Fig. 4*C*) nor the variability (gray areas Fig. 4*C*) of the reaction times changed over time for both monkeys and human listeners. This stable performance indicates the absence of perceptual learning during the experiments and permitted pooling of the data across different recording sessions.

### Ripple Detection Performance and Stimulus Variability

Figure 5 illustrates two psychometric response datasets, performance (% correct; Fig. 5*A*) and response latency (Fig. 5*B*), for one human (*h1*, *left*) and one monkey (*m1*, *right*) listener. Both responded to the same dynamic ripple (Ω = −3.0 c/o, ω = 32 Hz), presented under various modulation depths Δ*M* and randomized static noise durations *D*.

The estimated thresholds, Δ*M* at vertical midpoint between lower and upper bound of the black fitted curves (Fig. 5*A*) were comparable for the two listeners, as indicated by the crossings between the vertical and horizontal gray lines in Fig. 5*A* (*h1*: 27 [23–33]% vs. *m1*: 24 [20–31]%). The estimated slopes (β [95%-CI]), however, differed significantly (*human h1*: 3.5 [2.5–3.9] vs. *monkey m1*: 2.1 [1.1–2.4]).

The response reaction time decreased systematically with increasing Δ*M* (Fig. 5*B*). Here, the upper and lower limits (horizontal gray lines in Fig. 5*B*) of the fitted curves correspond to the peaks of hits and misses in Fig. 4*B*, respectively. Note, however, that other studies have found that for a given Δ*M* reaction time changes systematically as a function of ripple velocity as well as ripple density (18, 46). That is, the reaction time is determined by all the three parameters defining the amplitude envelope of a ripple stimulus: *1*) velocity ω (Hz): temporal modulation; *2*) density Ω (c/o): spectral modulation; and *3*) modulation depth Δ*M* (%).

Stimulus variability can be a confounding factor in the sense that longer delays in ripple onset, *D*, may induce a more liberal placement of the internal decision criterion, resulting in different response latencies (69). To check for this possible confound, we analyzed hit latency against *D* but did not obtain any systematic relationship (Fig. 5*C*). This was verified by Kendall’s rank correlation, one-tailed test: *h1*: tau-b < 0.1, *P* > 0.1 (Fig. 5*C*, *left*); *m1*: tau-b < 0.07, *P* > 0.2 (Fig. 5*C*, *right*). Comparable nonsignificant *P* values were obtained for the other listeners, *h2–h5* and *m2–m5*.

### Statistical Analysis Psychometric Parameters: Threshold versus Slope

The expected performance functions of the fitted psychometric curves (Fig. 5*A*) were parameterized as a cumulative Weibull distribution function *F*(*x*; α; β) (*Eq. 4*), wherein α determines the threshold and β determines the slope. Figure 6 summarizes an across-subject characterization of the fitted psychometric data, pooled across all combinations of ripple densities and velocities.

Figure 6*A* shows the probability density distributions of the α and β values, pooled across human (*h1–h5*, light shading) and monkey (*m1–m5*, dark shading) listeners, respectively. An across-subject analysis of the α or β distributions for testing within-species differences did not reveal any significant difference (2 sample: *n*_1_ = 87, *n*_2_ = 435; 1-tailed K-S statistic: *humans h1–h5* α: *k* ≤ 0.16, *P* > 0.1; β: *k* ≤ 0.12, *P* > 0.05 vs. *monkeys m1–m5* α: *k* ≤ 0.15, *P* > 0.1; β: *k* ≤ 0.18, *P* > 0.05). Next, we established that the species-specific α distributions (human vs. monkey) did not differ in overall shape either (2 sample: *n*_1_ = 435, *n*_2_ = 435, 2-tailed K-S statistic: *k* = 0.09, *P* > 0.05), as can be inferred from their corresponding cumulative distributions (Fig. 6*A*, *left*, *inset*).

In contrast, the slopes of the pooled monkey data were consistently lower compared with those of the pooled human data (Fig. 6*A*, *right*, *inset*): the peak of the human β probability density function is centered at 3.6 (bandwidth: 4.5) versus that of the monkeys at 2.6 (bandwidth: 2.4). K-S testing confirmed that these distributions were significantly different (2 sample: *n*_1_ = 435, *n*_2_ = 435, *k* = 0.44, *P* < 0.0001). Thus, ripple detection thresholds were determined with a higher discriminating power, i.e., steeper slopes, in humans than in monkeys. Importantly, the narrow bandwidths of β suggest that the ripple-based S-T sensitivity is characterized by a relatively constant slope of the psychometric curves.

In Fig. 6*B*, we compare the ripple thresholds of each listener with those pooled and averaged across humans (*h1–h5*; Fig. 6*B*, *left*) and monkeys (*m1–m5*; Fig. 6*B*, *right*), respectively. The large overlap between the 95% CIs of the squared correlation coefficients and their proximity to unity reveal a close correspondence between the averaged and the respective individual threshold data for both humans (Fig. 6*B*, *left*, *inset*) and monkeys (Fig. 6*B*, *right*, *inset*).

### Comparative Characterization of Raw Performance Data

**Figure 7.**
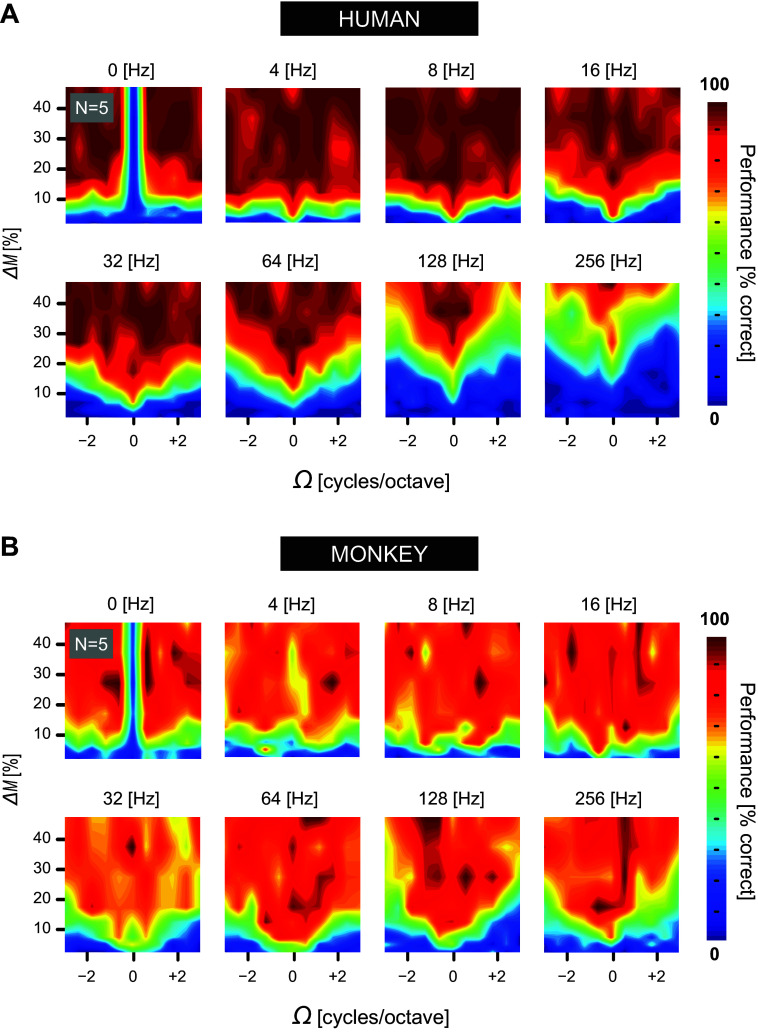
Performance characterization to dynamic ripples. Averaged contour performance plots of modulation depth (Δ*M*: 0 to 50%) against ripple density (Ω: −3.0 to +3.0 c/o) for all 8 ripple velocities (ω: 0 to 256 Hz). Performance levels are color coded. Within each subplot, isodensity lines represent raw psychometric functions, like the fitted ones in Fig. 5*A*. Contours were smoothed for illustrative purposes. *A*: pooled human data (*N* = 43,115 trials). *B*: pooled monkey data (*N* = 107,787 trials). See glossary for abbreviations.

Figure 7 provides a complete overview of the relationship between the raw (i.e., nonfitted) performance data and the S-T parameters of all dynamic ripple stimuli. Each colored contour plot shows a two-dimensional performance pattern for a particular ripple velocity, whereby the performance levels belonging to a unique (Ω,ω) combination are ordered vertically as a function of Δ*M*. We observed several striking similarities and differences between the pooled raw performance patterns of human (*h1–h5*; Fig. 7*A*) and monkey (*m1–m5*; Fig. 7*B*) listeners. First, the blue-yellow-colored contours shift progressively upward with increasing ripple velocity, ranging from 4 up to 256 Hz. Its progression, however, is more prominent in humans than in monkeys, signifying that monkeys are more sensitive, i.e., better performance at low modulation depths, to ripple velocities above 16 Hz. Second, performance decreases with increasing ripple density. This trend occurs in both species and is most prominent for the high velocities.

The human performance patterns, however, have dark red contours that are not seen in the monkey results. This demonstrates that monkeys rarely reached 100% performance for modulation values up to 50%, as was shown in Fig. 6*A* for *monkey m1*. Moreover, the human response patterns show less variability, i.e., clearer transitions in coloring. This characteristic is consistent with the observation that the averaged guess rate of the monkeys was higher than that of humans (see *Robustness of Detection Threshold Estimation* in appendix). Also note that the isodensity contours at 0 c/o (vertical midlines in Fig. 7) in the 0-Hz velocity plots are dark blue. Thus, the catch trials evoked adequate low performance levels in all listeners, thereby signifying their nonmodulated acoustic content.

Overall, the raw performance data of Fig. 7 agree well with the statistical analysis of the fitted psychometric data summarized in Fig. 6. Within the same species ripple detection performance shows a low degree of variability, whereas between species systematic differences may be observed.

### Comparative Characterization of the S-T MTF at Threshold

**Figure 8.**
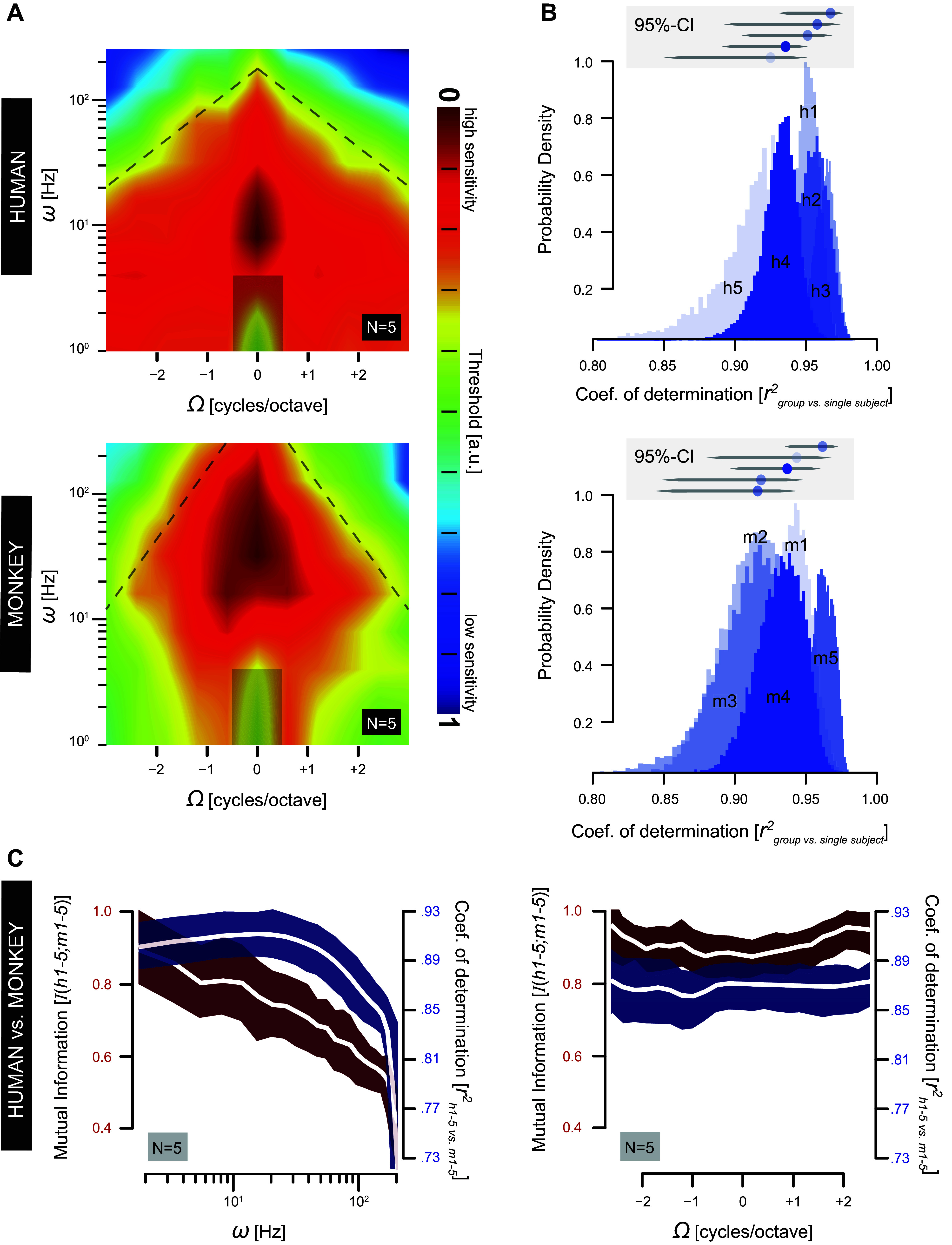
Comparative characterization of the S-T MTF. *A*: normalized MTFs based on pooled and averaged threshold data of human (*top*) and monkey (*bottom*) listeners. Vertical axis: ripple velocity; horizontal axis: ripple density. Thresholds are normalized (*Eq. 6*) and color coded (bar) for visualization purposes only. Dashed lines indicate the falloff in sensitivity at high ripple velocities. Contours are smoothed for illustrative purposes only. The transparent rectangles are inferred from the guess rates obtained from catch stimuli (Ω: 0 c/o, ω: 0 Hz). Note the high congruence in shape of the MTFs, despite a vertical shift of ∼1 octave, between the high sensitivity regions (dark red contours). a.u., Arbitrary units. *B*: squared correlation probability density distribution functions between normalized MTF as determined for each subject individually (humans: *h1–h5*; monkeys: *m1–m5*) and the respective group human or monkey MTF as shown in *A*. Color-coded histograms (shades of purple) show the variation of the squared Spearman rank correlation coefficient *r*^2^ across 100,000 bias-corrected percentile bootstrap samples, as obtained for each subject separately. The gray *insets* represent the 95% CIs of the respective color-coded distributions shown below. Note the extensive overlap and similarity in shape signifying a close relationship between the individual and the pooled MTFs. *C*: quantitative comparison between human and monkey MTFs. Mutual information (purple) and *r*^2^ (red) as a function of log velocity (*left*) and ripple density (*right*). Colored areas indicate 95% CI. Mutual information is scaled onto the [0,1] range for illustrative purposes only. Note, however, that *r*^2^ signifies a linear dependence, whereas mutual information measures general dependence (including nonlinear relations) (64, 70). Mutual information highlights that human sensitivity to temporal ripple modulation deviates significantly and in a consistent manner from that of monkeys. The latter is not evident for sensitivity to spectral ripple modulations. The squared correlation analysis does not signify this significant difference in sensitivity to ripple velocity between humans and monkeys. See glossary for abbreviations.

The threshold-based MTFs of Fig. 8*A* were obtained by pooling and averaging the normalized MTF matrix, *M*_norm_(Ω,ω) (*Eq. 6*; see also Fig. 1*C*), for all human (*h1–h5*; Fig. 8*A*, *top*) and monkey (*m1–m5*; Fig. 8*A*, *bottom*) listeners, respectively.

The MTFs can be best characterized as follows. First, both species reach their peak sensitivity (dark red contours in Fig. 8*A*) around zero density (−0.6 to +0.6 c/o human vs. −1.2 to +1.2 c/o monkey). Along the (vertical) temporal modulation axis, however, peak sensitivity is shifted toward higher ripple velocities in the monkey MTF (30–60 Hz) compared with the human MTF (6–20 Hz).

Second, the limit of the S-T modulation rate is expressed by the slope of the velocity-density sensitivity (dashed lines in Fig. 8*A*). The steepness of this slope, determined through linear regression of the 0.38 (yellow) contour, in the monkey MTF {107 [105–109] log (Hz/c/o)} is 1.8 times higher compared with the slope of the human MTF {60 [57–61] log(Hz/c/o)}. Note also that their respective offsets at zero density (as [95% CI] Hz) are shifted by almost one octave with respect to each other: (monkeys: 287 [284–291] Hz vs. humans: 163 [162–165] Hz).

Third, Fig. 8*B* shows the probability density distribution functions of the correlation coefficient between normalized MTF for each subject individually (humans: *h1–h5*; monkeys: *m1–m5*) and the respective averaged human or monkey MTF as shown in Fig. 8*A*. None of the distributions has a correlation coefficient lower than 0.8 and they overlap extensively (see gray *insets* in Fig. 8*B*), highlighting a close relationship between the individual and their respective pooled MTFs.

The main finding from the human and monkey threshold-based MTF is a systematically ordered but quantitatively dissimilar pattern of spectral-temporal modulation sensitivities. We used two distinct metrics on the MTFs to quantify the similarity of the two species statistically: mutual information (*Eq. 11*) and linear correlation. Figure 8*C*, *left*, emphasizes that for temporal modulation rates below 20 Hz the human and monkey MTFs do not differ significantly (purple: high correlation; dark red: high mutual information), whereas above 100 Hz the MTFs differ markedly. Figure 8*C*, *right*, shows the same measures plotted as function of the spectral modulation rate. Note that both the mutual information and correlation remain high and independent of ripple density, indicating that across ripple velocities the MTFs for monkeys and humans are highly similar in shape for all ripple densities tested.

### Testing for (In)Separability and Up/Down Symmetry of the S-T MTF at Threshold

**Figure 9.**
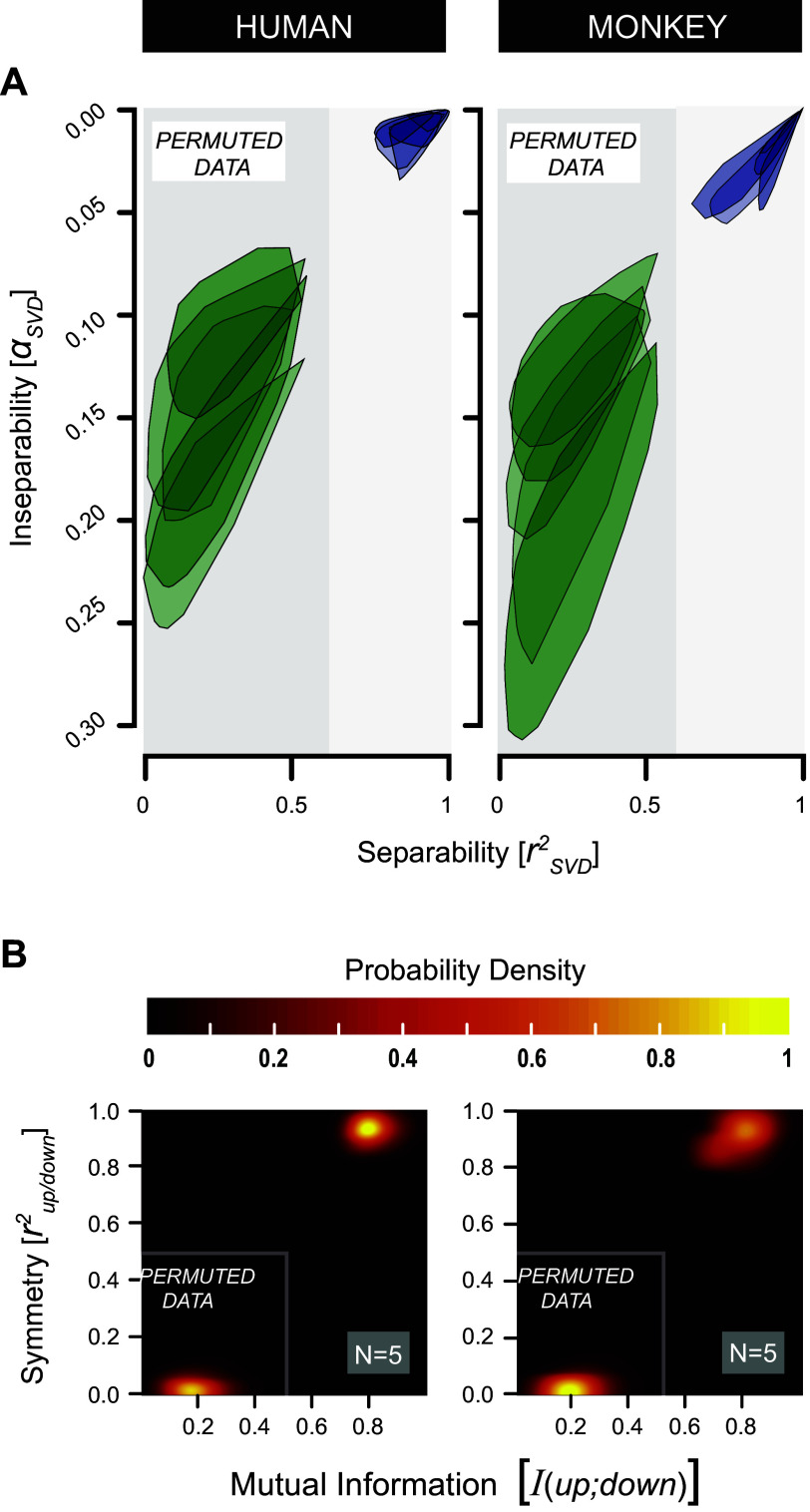
Intersubject separability vs. symmetry S-T MTF analysis. *A*: separability analysis: human (*h1–h5*; *left*) vs. monkey (*m1–m5*; *right*). Shown are inseparability (α_SVD_) vs. separability ($r_{\mathrm{SVD}}^{2}$) parameter plots. Each shaded area represents the convex hull enclosing 100,000 bootstrap samples, drawn from the original MTF data of single subjects. 95% CIs from randomly permuted MTFs (green) do not overlap with the those of the measured data (purple). The latter are positioned close to (1,0), in line with spectral-temporal separable MTFs (see Fig. 2 for explanation). *B*: up/down MTF symmetry analysis: human (*h1–h5*; *left*) vs. monkey (*m1–m5*; *right*). Shown are color-coded 2-dimensional (2-D) probability density plots, coefficients of determination $r_{\mathrm{up/down}}^{2}$ vs. mutual information *I*(up;down). Permuted data represent samples from randomly reshuffled MTF values. The highest densities of the permuted data cluster close to (0,0), which arise due to chance alone. By contrast, highest densities from the original human and monkey data cluster around (0.8, 0.9), indicative of nearly perfect symmetry. See glossary for abbreviations.

Figure 9*A* summarizes our statistical analysis on the inseparability indices derived from SVD analysis of the 10 threshold-based MTFs, one for each listener. Here, α_SVD_ reflects the degree of inseparability of the measured data, with zero corresponding to full separability across the entire S-T domain. The $r_{\mathrm{SVD}}^{2}$ statistic reflects the proportion of variance accounted for when assuming separability. We compared α_SVD_ to $r_{\mathrm{SVD}}^{2}$ by means of bootstrap resampling for the individual human (*h1–h5*; Fig. 9*A*, *left*) and monkey (*m1–m5*; Fig. 9*A*, *right*) listeners. In the perfectly separable case, the data would be concentrated at ($r_{\mathrm{SVD}}^{2}$,α_SVD_) = (1,0) as demonstrated in Fig. 2.

Despite small quantitative differences, the bootstrap analysis gave identical results. In all subjects, the processing of S-T modulations appears to be predominantly separable. Convex hulls corresponding to the measured data (purple in Fig. 9*A*) lie close to the (1,0) point and do not overlap at all with the simulated convex hulls determined by chance alone (green in Fig. 9*A*). The latter were generated by randomly permuted MTFs. Yet, the small, but systematic, deviations from the (1,0) point could be explained by the contribution of a small inseparable component in the MTFs.

Figure 9*B* compares the mutual information and correlation measures to assess up/down symmetry of the MTFs. In both monkeys and humans, the S-T sensitivity pattern for upward (Ω < 0)-moving ripples closely resembles the pattern obtained for downward (Ω > 0)-moving ripples. First, peak density (bright yellow in Fig. 9*B*) of bootstrap samples derived from the measured MTF data is centered at (0.95, 0.83), which is close to the ideal (1) point, signifying perfect up/down symmetry as demonstrated in Fig. 2. Second, the latter do not coincide with the peak densities that arise by chance alone (derived from permuted data) [white *insets* in Fig. 8*B* with the highest densities at (0.18, 0.04), which is close to (0, 0), the point representing a total absence of symmetry]. Third, despite the slightly higher variability of the monkey data, the peak densities of both species lie closely together.

**Figure 10.**
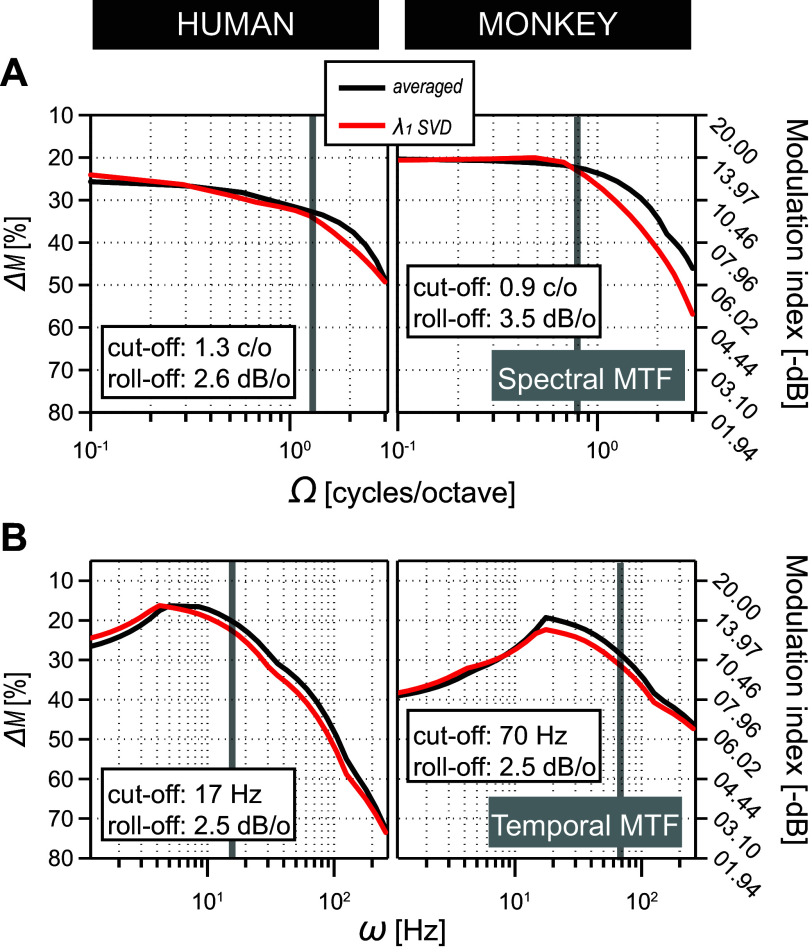
Comparative analysis of spectral vs. temporal MTFs at threshold. One-dimensional spectral (*A*) and temporal (*B*) MTFs of humans (*left*) and monkeys (*right*). Black lines represent the averages of the individual **G**_Ω_(ω) and **H**_ω_(Ω) vectors of either the pooled human (*N* = 5) or monkey (*N* = 5) MTFs of Fig. 8*A*. Red lines represent the 1st eigenvalue SVD reconstructions (*Eq. 7*) of the averaged data. Modulation index (*right y*-axis) is given by −20 × log_10_(Δ*M*/100) (in dB). Curves are smoothed for illustrative purposes only. Note that compared with humans the spectral and temporal modulation sensitivity of macaques is shifted to the lower end of the frequency domain and the higher end of the time domain, respectively. Gray lines: cutoffs of the respective SMTFs and TMTFs. Despite these cross-species differences, the close match between the (red) λ_1_-SVD reconstructed data and the (black) averaged data strongly supports full separability of S-T sensitivity in both humans and monkeys. See glossary for abbreviations.

In addition, we determined the first singular vectors from the SVD analysis to assess the general shape of the spectral (red curves, Fig. 10*A*), and temporal (red curves, Fig. 10*B*), MTF, **H**(Ω) and **G**(ω), respectively (*Eq. 7*). These λ_1-SVD_-reconstructed one-dimensional transfer functions were compared with the averages of the actual measured transfer functions (black curves, Fig. 10, *A* and *B*): note the close similarity in shape of the red and black curves. These results are consistent with the pooled and normalized MTFs of Fig. 8*A* and the intersubject MTF analysis of Fig. 9*A*. First, the threshold-based S-T MTF can be generally characterized as spectrally low pass and temporally band pass. Second, the separable portion of the threshold-based S-T MTF is a viable descriptor of the original data.

Although Figs. 9 and 10 convincingly demonstrate that the spectral-temporal MTFs of humans and monkeys are strongly governed by separable spectral and temporal processing mechanisms, small but systematic deviations from the ideal separability points at (1,0) and (1) were also noticeable in Fig. 9. These deviations could be largely explained by including the contribution of a small inseparable component in the MTFs, through the second singular value, λ_2_. To demonstrate this, we compared the reconstructed MTFs from the separable analysis [*Eq. 7*, with *K*(λ*_i_*) = λ_1_, i.e., a scalar] with the reconstruction that included the first two singular values {*K*(λ*_i_*) = [λ_1_ 0; 0 λ_2_], i.e., a 2 × 2 diagonal matrix}.

**Figure 11.**
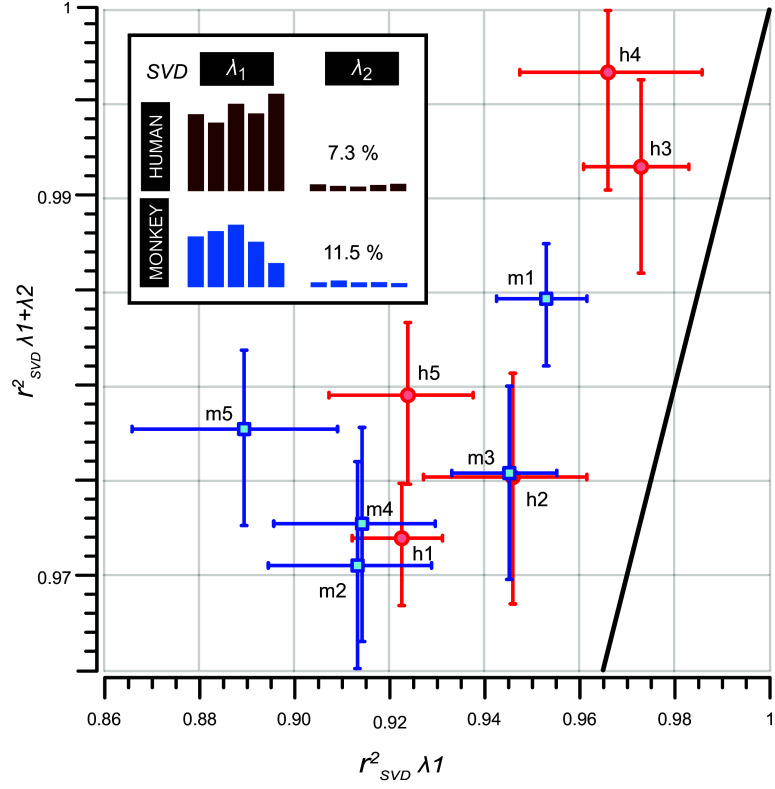
Relative contribution of singular values to the 2-dimensional (2-D) shape of the S-T MTF. Shown is a $r_{\mathrm{SVD}}^{2}$ λ_1_ vs. $r_{\mathrm{SVD}}^{2}$ λ_1_ + λ_2_ scatterplot comprising MTF data of all human (red, *h1–h5*) and monkey (blue, *m1–m5*) listeners. All plotted coefficients of determination were calculated by performing a Spearman’s rank correlation between each of the 88 elements of *M*_rec_(Ω,ω), reconstructed from either the 1st SVD eigenvalues or, alternatively, the SVD of 1st + 2nd eigenvalues, and the original *M*(Ω,ω). The vertical and horizontal error bars represent 95% CIs. The diagonal (black) corresponds to the identity line. The 2nd eigenvalue of all the measured S-T MTFs adds a small, but consistent, contribution to the reconstructed MTF_rec_. Note that the averaged λ_2_ is ≈7% and ≈11% of the ${\bar{\lambda }}_{1}$ values for human (brown bars) and monkey (blue bars) listeners, respectively. This is illustrated in the *inset* (*top left*). In other words, the first singular value, λ_1_, can be used to recover the overall 2-D shape of the original MTF for up to 95% in humans and 90% in monkeys. Apparently, there is a significant inseparable component to the S-T sensitivity tuning in both species. See glossary for abbreviations.

Figure 11 compares the $r_{\mathrm{SVD}}^{2}$ coefficients of determination for the two reconstructions for each individual listener. Although the inclusion of only the first singular value already accounted for >93% of the variability in the reconstructions for every participant (horizontal dimension, Fig. 11), adding the second singular value improved the reconstruction for all individuals, as now all $r_{\mathrm{SVD}}^{2}$ > 0.98 (vertical dimension, Fig. 11). Interestingly, this result held equally for the human and monkey participants. This leaves open the possibility that apart from a dominant fully separable S-T processing mechanism in the primate auditory system, there is a small but consistent contribution from an inseparable mechanism as well. Indeed, the relative value λ_2_/λ_1_ varied between 5.1% and 8.8% for the humans and between 7.7% and 17.0% for the monkeys. Including also the third singular value led to a minor, just significant, further improvement of the fit (not shown).

### Comparative Characterization of the S-T MTF at Suprathreshold

**Figure 12.**
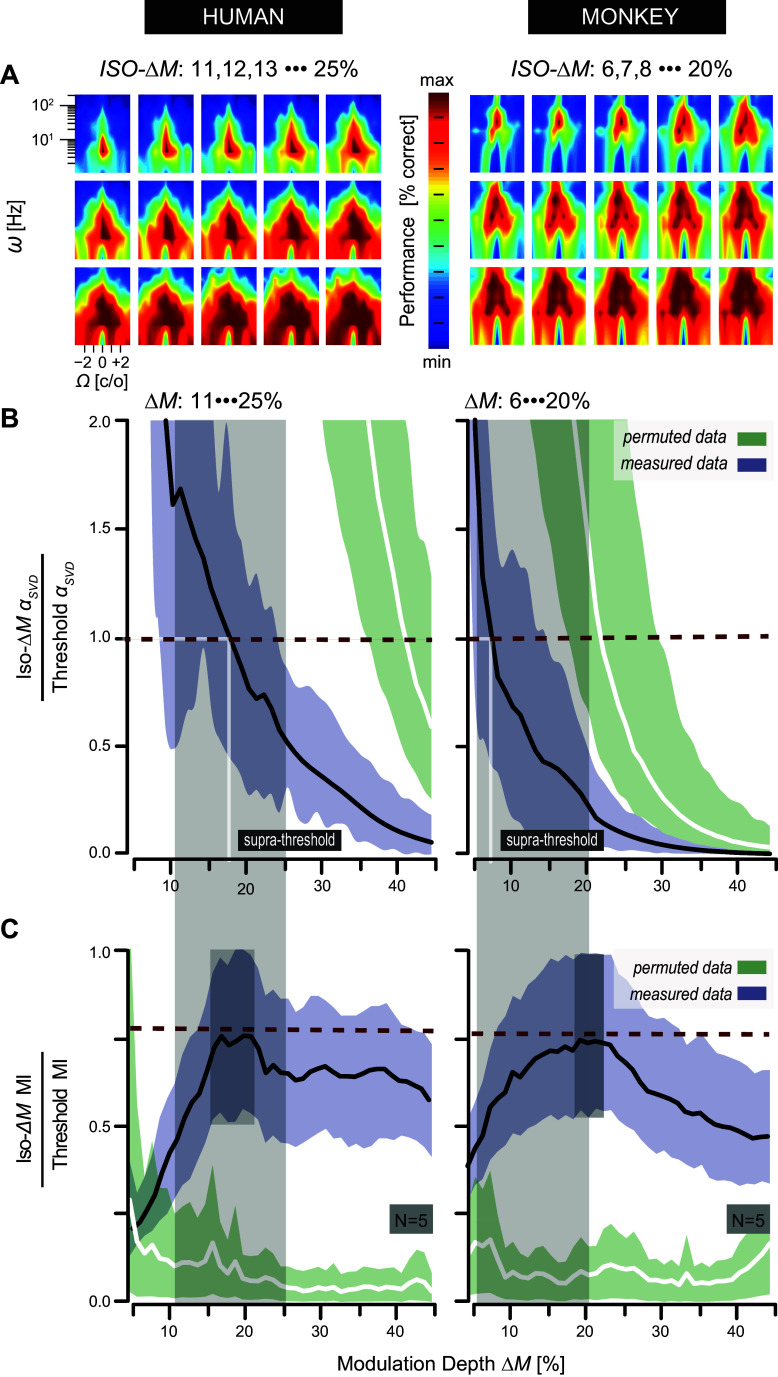
Suprathreshold characterization of the S-T MTF. *A*: incremental Δ*M* chronology of 15 iso-Δ*M* MTF contour plots for human (Δ*M*: 11–25%; shaded areas in *B* and *C*, *left*) and monkey (Δ*M*: 6–20%; shaded areas in *B* and *C*, *right*) listeners, respectively. Each colored plot represents the pooled and averaged data of human (*left*, *N* = 5) or monkey (*right*; *N* = 5) listeners. Color code represents performance level (% correct). Contours were normalized and smoothed for illustrative purposes only. Note the striking 2-dimensional (2-D) shape congruency of many iso-Δ*M* plots with the threshold contour plots of Fig. 8*A*. *B*: normalized iso-Δ*M* MTF α_SVD_ plotted as a function of Δ*M* (black lines). 95% CIs from randomly permuted MTFs (green) do not overlap with the measured data (purple), except for monkey Δ*M* values above 40%. Data are normalized to facilitate comparison with threshold-defined MTFs. Because of normalization, abscissa values < 1 (dashed red lines) denote iso-Δ*M* MTFs with a lower degree of separability as was estimated for the corresponding MTFs at threshold. Note that at unity (cross section, vertical white lines), however, the iso-Δ*M* MTFs mirror the threshold MTF in terms of their inseparability index. Normalized separability indices at unity occur at Δ*M* ≈ 17% (human) and ≈ 7% (monkey). *C:* normalized iso-Δ*M* mutual information (MI) is plotted as a function of Δ*M* (black lines). 95% CIs from randomly permuted MTFs (green) do not overlap with the measured data (purple), except for Δ*M* values below 10%. Data are normalized to facilitate comparison with threshold-defined MTFs. Because of normalization, maximized MI equals unity. That is, iso-Δ*M* MI was divided by the maximal obtainable MI at threshold. The latter was obtained for each listener by computing the mutual information between identical pairs of threshold-MTFs. Dark gray shaded areas denote the Δ*M* values for which the respective iso-Δ*M* MTFs are comparable to the threshold-MTF: Δ*M* ∼16–21% (humans) vs. Δ*M* ∼18–23% (monkeys). See glossary for abbreviations.

So far, we have constrained our analysis to the perceptual detection thresholds of dynamic ripples. Here, we examine the important question of to what extent the threshold-based MTFs generalize to the “ineligibility” of clearly audible, suprathreshold, ripple modulation depths (for explanation see Fig. 1*B*). Figure 12 summarizes our suprathreshold analysis of the S-T MTF.

Figure 12*A* shows a subset of the iso-Δ*M* MTF contour plots for Δ*M* =11–25% (human, *N* = 5) (*h1–h5*) and Δ*M* = 6–20% (monkey, *N* = 5) data. Note the systematic changes in both the human (Fig. 12*A*, *left*) and monkey (Fig. 12*A*, *right*) iso-ΔM MTF chronology. First, the regions of higher performance levels (red coloring in Fig. 12*A*) gradually increase in size as function of Δ*M*. Second, irrespective of its size, the overall shape of this region appears to be conserved up to Δ*M* levels that supersede the measured thresholds for most stimuli (cf. Fig. 6*A*, *left*).

In Fig. 12*B* we quantify the shape of the iso-Δ*M* MTFs, up to 45% modulation depth, well above threshold, with respect to the threshold-based MTFs of Fig. 8*A* in terms of their full separability. Note that a value below 1.0 (red dashed lines in Fig. 12*B*) indicates a higher degree of separability at suprathreshold than was the case for the threshold MTFs. Despite quantitative differences, the iso-Δ*M* MTF analysis shows that both the human and monkey auditory systems preserve S-T separability and direction sensitivity symmetry at suprathreshold levels.

In Fig. 12*C*, the ranges of maximal mutual information for stimulus modulation levels between Δ*M* ∼ 16–21% in humans and between Δ*M* ∼ 18–23% in monkeys compare well to the values obtained from the threshold MTFs, indicating that the overall 2-D shape of the MTF is preserved for these suprathreshold stimuli.

## DISCUSSION

Psychoacoustic measurements of perceptual detection thresholds to dynamic ripples have so far been performed only in humans and songbirds (38, 40) and have not included suprathreshold analyses. Here we report on the perceptual spectrum/time sensitivity to inseparable naturalistic acoustic stimuli in normal-hearing humans and rhesus monkeys across their dynamic hearing range. Using the same psychophysical methods for both species, we collected free-field hearing thresholds to pure tones and a large dataset of manual reaction times for the full range of audible spectral-temporal modulations. Together, these results provide a unique database to assess and compare the hearing capabilities of human and nonhuman primates for near-threshold and above-threshold sensation levels. The data show that rhesus monkeys have poorer low-frequency and superior high-frequency hearing than humans (Fig. 3), confirming previous reports (68), and a higher sensitivity to temporal modulations >100 Hz than humans (Figs. 7, 8, and 10), as reported for pure temporal modulations (51).

Our central new finding is that in both species spectral-temporal processing is well understood by largely independent unbiased contributions from two separable components: the averaged spectral *H*_ω_(Ω) and the averaged temporal *G*_Ω_(ω) modulation transfer functions (black curves, Fig. 10), respectively. These two components could explain >90% of the response data (Fig. 11). This finding not only held near the modulation detection threshold but extended to suprathreshold modulation depths as well (Fig. 12). Interestingly, a significant contribution from inseparable, processing channels, in which neural populations are tuned to ripples, was also identified for all individuals of both species (Fig. 11).

### Psychoacoustics

By applying dynamic ripples that cover the S-T sensitivity range, we avoided testing humans and monkeys to an arbitrary, and possibly biased, set of biological sounds (e.g., conspecific vocalizations) or natural sounds (e.g., recorded environmental noises). Our approach deviates from previous psychoacoustic studies (11, 38–40, 71–74) in that listeners were exposed to a large variation in the stimulus parameters while determining complete psychometric functions for 87 different spectral-temporal (Ω,ω) combinations (Fig. 1*C*). Studies that applied S-T modulated sounds determined threshold but not suprathreshold performance (38–40) or discrimination performance (25–27, 75) on a limited set of S-T combinations.

In our study, listeners could never predict which ripple to expect. As such, they could only respond consistently to the sound when attending to the S-T amplitude modulations, rather than to some random spurious event that could have been present in the transition from static noise to ripple. The consistent reaction time distributions (Fig. 4), the low across-subject response variability for both humans and monkeys (Figs. 6 and 7), and the consistent misses for catch trials (Fig. 7) confirm the validity of our experimental approach.

### S-T MTFs versus MPS of Speech

**Figure 13.**
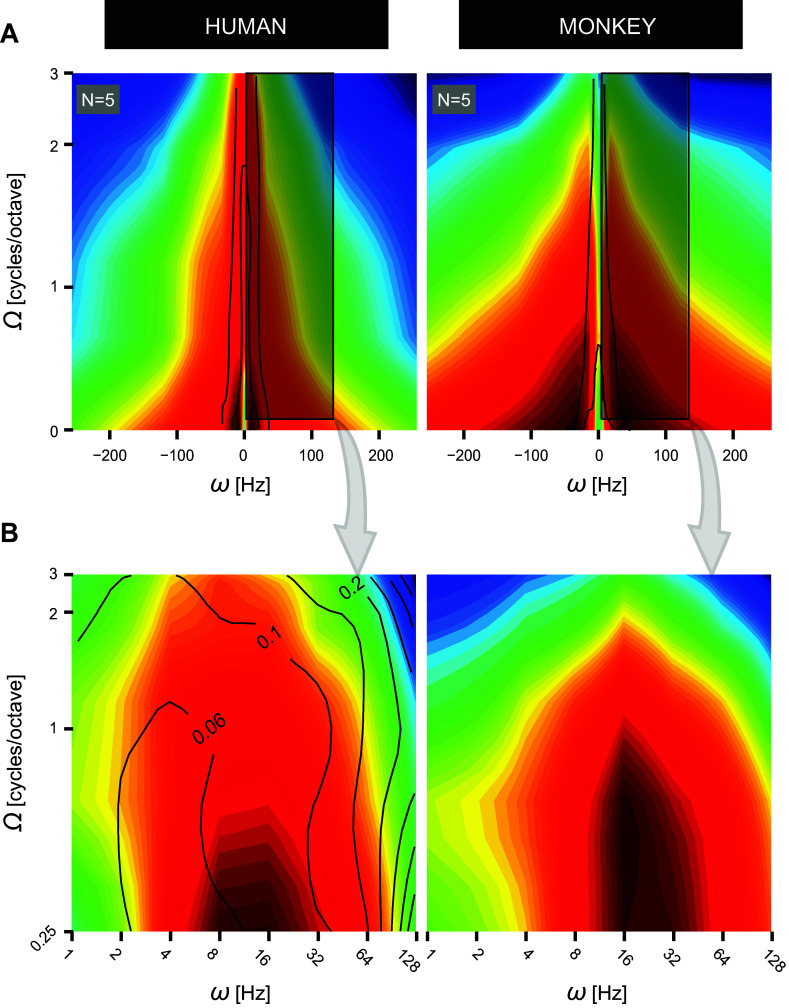
S-T hearing in humans and monkeys compared to known human modulation power spectrum (MPS) data. *A*: S-T MTF results of the present study (colored contours) remapped in a format equivalent to that reported by Elliott and Theunissen (11) for the MPS of male speech. The MPS forms a second-order representation derived from spectrogram analysis, which is commonly used to visualize and quantify the joint S-T modulations of human speech. For comparative analysis, we show the remapped data of the monkey MTF as well. *B:* subset of the data (shaded areas) shown in *A* in a format equivalent to that reported by Chi et al. (38). The overlaid black isoperformance lines (*left*) represent the data from Chi et al. (38). See glossary for abbreviations.

Figure 13*A*, *left*, provides a direct comparison of human S-T hearing from our MTF data (Fig. 8*A*, *top*) with the reported human modulation power spectrum (MPS) of speech (11). From the overlaid outer and inner contour lines, delineating the 90% and 95% of the modulation power in male (American English) speech, it is obvious that the ripple-based S-T window in humans extends well beyond the dominant modulation spectra of speech. Unfortunately, a direct comparison of our monkey MTFs with the MPS of their vocalizations (12) is not possible, as the ripple density of available MPS is provided in cycles per kilohertz, whereas our MTFs were based on ripples with logarithmic density (cycles/octave). Irrespective of this scaling difference, the monkey vocalization MPS correlates strongly with that of human speech: *r* = 0.82 (12). We thus expect that the monkey MTF (Fig. 13*A*, *right*) is likely to extend beyond the dominant modulation spectra of its vocalizations too.

When comparing our results (colored contours, Fig. 13*B*, *left*) to the psychophysical MTF data from Chi et al. (38) (black contour lines), it is clear that in both studies the perceptual MTF can be characterized as temporal band pass and spectral low pass. The only apparent difference is that the highest sensitivity in our MTF is observed at higher temporal modulations (dark red area around 10 Hz in Fig. 13*B*, *left*) than in the monkey study (38) data (centered around 4 Hz within the 0.06 contour line). The study in Ref. 11 applied an alternative filtering method, closely related to the use of ripples, from which they derived the S-T MTF for speech intelligibility. They compared their data with the psychoacoustic MTF from Chi et al. (38), showing a high degree of similarity. Given the considerable methodological differences, the similarity in shape between respective MTFs suggests that ripple-based MTFs provide a robust objective measure for S-T hearing in humans. Finally, it should be noted that despite the shift toward higher frequencies of the temporal modulation axis, the monkey MTF (Fig. 13*B*, *right*) bears considerable resemblance to the human MTF (Fig. 13*B*, *left*).

### Spectral versus Temporal Modulation

Here we discuss how the dissection of the MTF into one-dimensional spectral and temporal modulation transfer functions (Fig. 10) compares to earlier measurements of pure spectral, here denoted by *H*_0_(Ω), or pure temporal *G*_0_(ω) transfer functions, respectively. From the data (black curves) in Fig. 10 it can be seen that our averaged SMTFs (Fig. 10*A*) and TMTFs (Fig. 10*B*) corroborate the band-pass/low-pass characteristics as is typically found in comparative studies on vertebrate hearing (76).

*H*_0_(Ω)-defined SMTFs generally show a low-pass characteristic with comparable cutoff frequencies. Spectral modulation detection is most sensitive from 0.5 up to 3 cycles/octave, with a rolloff of ∼3 dB per octave. *G*_0_(ω)-defined TMTFs generally display a band-pass filter characteristic with a pronounced decrease in sensitivity at very low-frequency modulations (<3 Hz). Temporal modulation detection is most sensitive from 2 up to 20 Hz with a rolloff of ∼3 dB per octave. Moreover, the high consistency among our averaged TMTFs (*Macaca mulatta*) and the *G*_0_(ω)-defined TMTFs reported by Moody (73) (*M. fuscata* and *M. mulatta*) confirms that manual reaction times to dynamic ripples provide a robust objective measure of S-T sensitivity in monkeys.

### Threshold versus Suprathreshold S-T Hearing

The majority of meaningful biological sounds are well above hearing threshold (42, 77). Thus, it is not self-evident that the threshold-based MTF (Fig. 8, *A* and *C*) provides an adequate description of how the auditory system processes S-T amplitude modulations in general. Nor is it self-evident that dynamic ripples, covering the full S-T perceptual range, are processed approximately linearly over a wide range of modulation-depths. In particular, under many conditions linear models cannot account for cortical responses of the vertebrate auditory system to ripples (13, 28, 31, 78). It is therefore of particular relevance to determine how dynamic ripples are perceived at suprathreshold modulation depths (Fig. 12).

At suprathreshold Δ*M* values between Δ*M* ∼16% and 21% in humans and between Δ*M* ∼18% and 23% in monkeys, the respective iso-Δ*M* MTFs closely match the 2-D shape of threshold-MTFs (Fig. 12*C*). This result may be due to the approximately constant slope of the psychometric curves across ripple modulations, which resulted from our statistical analysis of estimated slopes (β) of the Weibull curves (Fig. 6*A*), whereas 85% of the thresholds (α) were at Δ*M* < 20%.

A representation of independent spectral and temporal processing in threshold and suprathreshold acoustic regimes may explain why the S-T window of hearing in humans and macaques extends beyond the dominant modulation spectra of their own (conspecific) vocalizations (11, 12). That is, if S-T processing puts a premium on the statistical properties of natural sounds to obtain an efficient representation of spectrum and time (10, 43, 44), it is to be expected that hearing does not show an obvious perceptual bias toward particular (e.g., conspecific) sounds. This unifying hypothesis of S-T hearing in humans and monkeys goes against the specialization hypothesis that the auditory brain is specifically adapted to represent speech or vocalizations, to explain why animal brains are better at representing conspecific vocalizations than those of other animals (79). Secondary adaptations to specific behaviorally relevant sounds, leading to a hybrid hypothesis, are discussed in the following section.

### (In)Separability

The presence of both separable and inseparable S-T neurons in the auditory processing stream may underlie our finding that spectrum-time auditory perception is nearly separable (Figs. 8*A*, 9, and 10) but still shows an additional inseparable characteristic (Fig. 11). We also found that the S-T MTFs of humans and monkeys are to a large extent spectrum/time independent, with no preference for either upward or downward moving ripples (as illustrated in Fig. 2, *top*). The dominant separable system may be expressed in separate rostral and caudal streams (80) and/or along right and left hemispheric differences in spectral and temporal sensitivity, respectively (2). Our data thus suggest that the auditory system may primarily process sounds through independent channels, corresponding to a separable system that represents the spectral and temporal eigenvectors of Fig. 10, and complemented by an inseparable system, which accounts for a higher sensitivity to S-T modulations. Neurophysiological recordings suggest a systematic increase in the percentage of inseparable STRFs of auditory neurons from midbrain IC to primary auditory cortex (28–36). Thus, although sound processing could occur in a separable way at an early level [say, up to the IC (31, 32, 36)], inseparable S-T filters can still be present at higher areas and reflect their contribution to the percept as an inseparable component. Although it may seem wasteful to have both separable spectral and temporal filter banks and tuned S-T filters, it should be kept in mind that a limited set of such filters may represent a vast acoustic world (81).

Furthermore, separable and inseparable neural processing streams allow the system to flexibly develop highly selective and adaptive tuning to specific behavioral needs (e.g., attending to conspecific vocalizations in the presence of background masking sounds) without interfering with overall (separable) spectral and temporal processing and sensitivity. In this way, the perceptual response to the acoustic environment can rapidly adjust its sensitivity to meet task requirements or optimization needs in complex, unpredictable acoustic scenes. Adaptation to new context has been demonstrated in several behavioral animal experiments (1, 82, 83) and in humans, even specifically for spectrotemporal modulations (75).

### Audition versus Vision

Several studies (21, 37, 82–87) have fueled the idea that the primary auditory cortex responds to dynamic ripples in a way that is analogous to responses of primary visual cortex to moving visual gratings. The point of view adopted is a representation of natural sounds and images that is consistent with efficient statistical principles to extract features of fundamental importance to the respective sensory systems (88–91). The implication is that at the cortical level the behaviorally relevant acoustic attributes of dynamic ripples, spectral modulation frequency, temporal modulation frequency, modulation depth, are represented in much the same way as those of moving visual gratings, i.e., spatial frequency, temporal frequency, luminance contrast.

A clear indication of how natural stimuli are encoded within the auditory and visual systems may be found in the way they process spectrum-time versus space-time, respectively. Equivalence within these sensory systems would then be expressed by their (in)separability. Note that separability is a continuous variable (see *Eq. 8* and Fig. 2), and the degree of separability depends strongly on the metric used to represent the data (38, 92).

**Figure 14.**
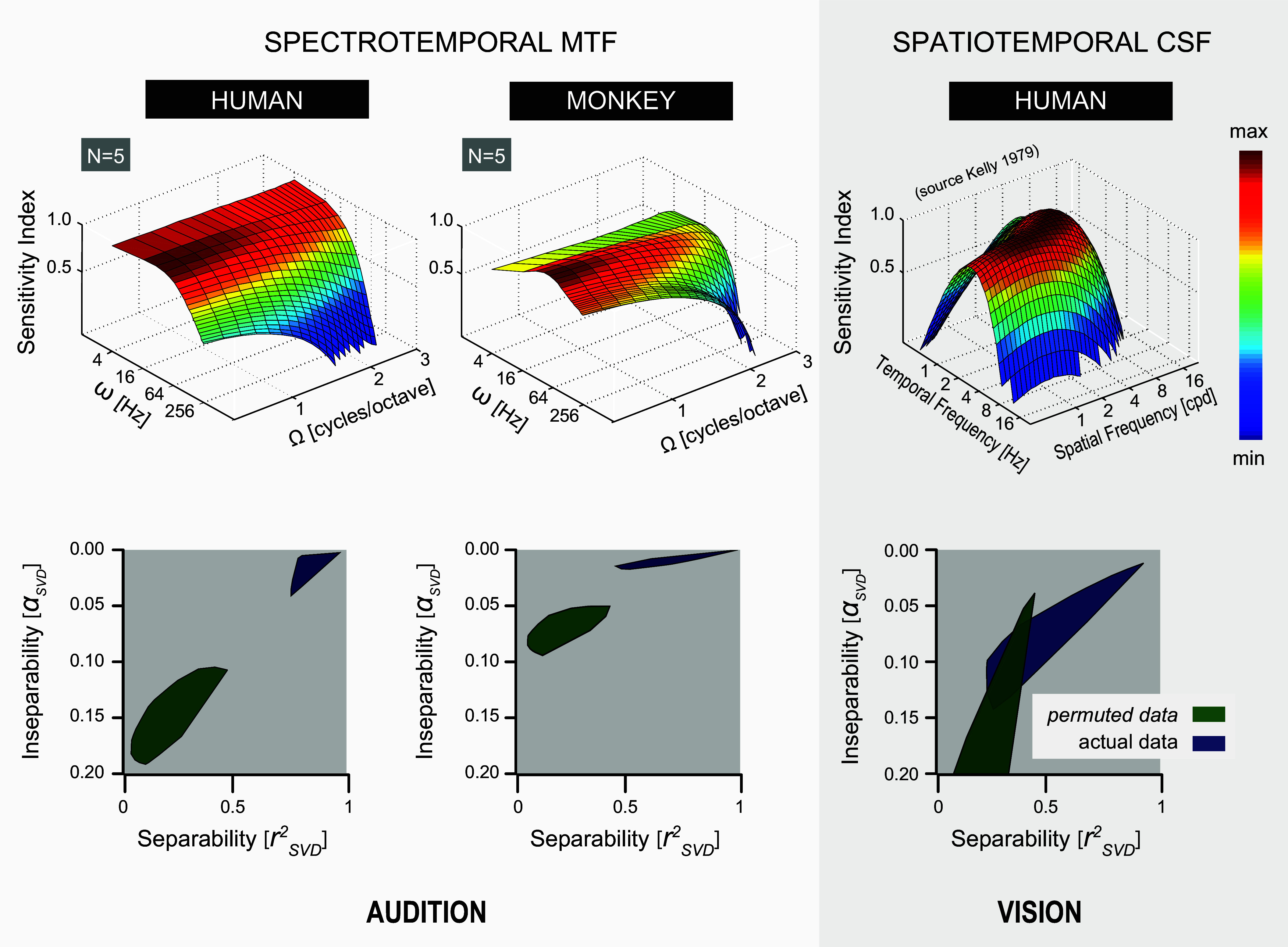
Frequency-time hearing vs. known human space-time vision. *Top left*: the single surfaced shapes derived from human audition (*left*) and monkey listeners (*right*) represent their averaged auditory MTFs, which define the frequency-time window of S-T hearing. We used a 5th-order 3-dimensional (3-D) polynomial model to describe the S-T MTFs. The coefficients representing the relationship between the fitted data points of the model are in provided in Table A1. *Top right*: for comparative analysis (vision), we show the human spatiotemporal contrast sensitivity function (CSF), defining the space-time window of vision [data from data from Kelly (45), with permission; see *Visual Spatiotemporal MTF Modeling* in appendix]. The vertical axis of an auditory MTF represents the depth modulation sensitivity index, which is the reciprocal of the minimum perceptual amplitude modulation required to detect a rippled noise. The axes of a visual MTF, however, represent the spatial and temporal frequencies of a contrast-reversing pattern and the observer's contrast sensitivity. Color encodes isosensitivity regions with a resolution of 10%, ranging from light blue (10–20%) up to dark red (90–100%). *Bottom*: inseparability (α_SVD_) vs. separability ($r_{\mathrm{SVD}}^{2}$) parameter plots in the same format as Fig. 9*A* but now for normalized and pooled S-T MTF data, as shown in Fig. 8*A*. Note the prominent overlap between permuted (green convex hulls) and actual (purple convex hulls) data for the visual MTF, indicating inseparability of space and time in perceptual human vision. See glossary for abbreviations.

To provide a fair assessment of visual and auditory sensitivity to naturalistic stimuli we therefore performed identical statistical tests on two separability indices, α_SVD_ and $r_{\mathrm{SVD}}^{2}$, to compare separability of the spatial-temporal visual CSF (45) with the auditory S-T MTF. Figure 14, *top*, shows the sensitivity surfaces for audition (Fig. 14, *top left*) and vision [Fig. 14, *top right*; data from Kelly (45), with permission]. The auditory plots run nearly parallel to the spectral-temporal axes, which is in line with a dominant spectral-temporal separability (cf. Fig. 2). The visual data, however, indicate a clear oblique orientation of the spatial-temporal sensitivity surface, which suggests strong inseparability, as reported previously (93, 94). This is further corroborated by the bootstrap analysis of the separability indices (Fig. 14, *bottom*), which show that the randomly permuted visual MTF (green convex hull, Fig. 14, *bottom right*) overlaps considerably with the measured CSF (purple convex hull, Fig. 14, *bottom right*). In other words, the original data do not deviate significantly from those that arise by chance alone. This statistical analysis demonstrates that vision is space-time inseparable to a much larger degree than S-T sensitivity of audition. Thus, the specificity with which the auditory brain encodes natural sounds may be less stringent than the specificity needed to adequately deal with natural images.

## GLOSSARY

CI: Confidence interval
c/o: Cycle per octave
CSF: Contrast sensitivity function
*D*
: Stimulus duration
FM: Frequency modulated
*G*(α*,ν*): Space-time MTF model (*Eq. A3*)
*G*_Ω_(ω): Temporal MT (*Eq. 7*)
*h1–h5*
: Human listeners 1–5
*H*_ω_(Ω): Spectral MTF (*Eq. 7*)
*I*(*A;B*): Mutual information (*Eqs. 9*, *10*, and *A1*)
*K*(λ*_i_*): Singular eigenvalue matrix (*Eq. 7*)
*m1–m5*
: Monkey listeners 1–5
*M*4: normalized 4th moment *S*(*t*) (*Eq. 3*)
MI: Mutual information
MPS: Modulation power spectrum
MTF or *M*(Ω,ω): Modulation transfer function
*M*_norm_(Ω,ω): Normalized MTF (*Eq. 6*)
*M*(*x,y*): Spectro-temporal MTF model (*Eq. A2*)
*P*(*x;*α*;*β*;*γ*;*λ): Psychometric function (*Eqs. 4* and *5*)
RMS: Root mean square
*R*(*t,x*): Sinusoidal ripple amplitude envelope (*Eq. 2*)

$r_{SVD}^{2}$

: Statistic measure of MTF separability

$r_{up/down}^{2}$

: Statistic measure of up/down MTF symmetry
SMTF: Spectral MTF
SPL: Sound pressure level
S-T: Spectro-temporal
*S*(*t*): Ripple stimulus equation (*Eq. 1*)
SVD: Singular value decomposition
TMTF: Temporal MTF
α: Threshold
α_SVD_: Inseparability index (*Eq. 8*)
β: Slope
γ: Guess rate (false positives)
Δ*M*: Modulation depth (%) (stimulus strength psychometric curves)
λ: Lapse rate (misses)
ω: Ripple velocity (Hz): temporal modulation frequency
Ω: Ripple density (c/o): spectral modulation frequency

## DATA AVAILABILITY

Data will be made available upon reasonable request.

## GRANTS

This research was supported by the Dutch Organization for Scientific Research (NWO) ALW/VICI grant 865.05.003 and by the program “AI & Ethiek” Rotterdam University of Applied Sciences (R.v.d.W.), by NWO-ALW grant 809.37.002 (H.V.), and by a grant from the NWO-TTW Open Technology Programme 2022-8 [“Otocontrol-2.0”, nr. 20414 (A.J.v.O.)]

## DISCLOSURES

No conflicts of interest, financial or otherwise, are declared by the authors.

## AUTHOR CONTRIBUTIONS

R.v.d.W., H.V., and A.J.v.O. conceived and designed research; R.v.d.W. and H.V. performed experiments; R.v.d.W. analyzed data; R.v.d.W., H.V., and A.J.v.O. interpreted results of experiments; R.v.d.W. prepared figures; R.v.d.W. drafted manuscript; R.v.d.W., H.V., and A.J.v.O. edited and revised manuscript; R.v.d.W., H.V., and A.J.v.O. approved final version of manuscript.

## APPENDIX

### Robustness of Detection Threshold Estimation

To monitor the accuracy with which each detection threshold could be estimated throughout the recording sessions, we calculated their respective 95% CIs and displayed this measure as a function of cumulative trial number on a log-log scale. Figure A1 shows that the accumulation of data from subsequent recording sessions improved the estimates of the extracted thresholds in both humans (Fig. A1, *left*) and monkeys (Fig. A1, *right*). Note that the data shown cover the last 14,080 trials of each monkey and the last 7,040 trials of each human listener.

Compared with humans (≈8,600 on average), we needed 2.5 times as many responses from the monkeys (≈21,600 on average) to converge to a stable 95% CI below 10%. A possible source for this difference is the monkeys’ higher guess rate (26% vs. 4%), along with a much greater proportion of catch trial stimuli needed to keep the monkeys under stimulus control (35% vs. 15%).

Artificially reversing the chronology with which the data were obtained did not alter this result (Fig. A1, *insets*). This confirms that procedural and perceptual learning did not influence performance over time (48). Instead, the data show that the improvement in estimated thresholds over time results from an increase in the total number of responses per threshold estimation.

### Mutual Information Computation

To compute the mutual information measure *I*(*A*;*B*) from a pair of spectral-temporal **M**(Ω,ω) matrices, we constructed a joint histogram (64), defined as a function of two variables, with *A* = **M**_1_(Ω,ω) and *B* = **M**_2_(Ω,ω). To obtain *h*, the values of *A* and *B* were mapped onto the [*A*_min_, *A*_max_] and [*B*_min_, *B*_max_], range, respectively, with equally spaced bins as determined through the interpolation algorithm of Chen and Varshney (70) (for review see Ref. 95). The joint probability function used in the calculation of *I*(*A*;*B*) of a given **M**(Ω,ω) pair was then obtained by normalizing *h*:

(*A1*)
$$I\left(A;B\right)=\{\begin{array}{c} \sum_{a\in A}\sum_{b\in B}p_{A,B}\left(a,b\right)\cdot \log\;\frac{p_{A,B}\left(a,b\right)}{p_{A}\left(a\right)p_{B}\left(b\right)}\\ \text{with}\;p_{A,B}(a,b)=\frac{h\left(a,b\right)}{\sum_{a\in A,b\in B}h\left(a,b\right)}\\ p_{A}(a)\;=\sum_{b\in B}p_{A,B}\left(a,b\right)\;\;\\ p_{B}(b)\;=\sum_{a\in A}p_{A,B}\left(a,b\right)\; \end{array}$$

Mutual information between a pair of MTFs is maximal when they have identical shapes (i.e., linearly dependent), whereas *I*(*A*;*B*) is 0 if the MTFs are completely dissimilar.

To test the statistical significance of *I*(*A*;*B*) > 0, the mutual information values computed from the actual **M**(Ω,ω) measurements were plotted against those computed from randomly permuted but shuffle-corrected (70) versions of **M**(Ω,ω) (see Fig. 10*B*). This was achieved by generating 100,000 bias-corrected percentile bootstrap samples of *I*(*A*;*B*) for both the actual and randomized data (61).

### Auditory Spectrotemporal MTF Modeling

The human and monkey S-T MTF surfaces (audition: Fig. 14, *top left*) were approximated by fitting a fifth-order three-dimensional (3-D) polynomial model to the normalized MTFs shown in Fig. 8, applying the surface fitting tool (poly55) of MATLAB (The MathWorks, Inc.).

The coefficients of the 3-D surfaces were found for the following polynomial equation, with *x* = ω (in Hz) and *y* = Ω (in cycles/octave):

(*A2*)
$$M(x,y)=\sum_{\begin{array}{c} n,m\;=\;0\\ n\;+\;m\;\leq \;5 \end{array}}^{5}p_{nm}\cdot x^{n}y^{m}$$

The two sets of 21 coefficients are provided in Table A1. Note that the fitted surfaces only represent the modulation thresholds for downward-moving ripples. The data were smoothed by a factor of 10 through bivariate linear interpolation for illustrative purposes only. Finally, to ensure a fair assessment of the degree of inseparability of audition versus vision, the dimensions of the matrices defining the S-T MTFs (5 × 8) were identical to those defining the spatiotemporal visual MTF (5 × 8). Goodness-of-fit statistics: sum of squares due to error < 5; *R*^2^ > 0.88.

### Visual Spatiotemporal MTF Modeling

The human space-time CSF surface *G*(α,ν) of Fig. 14 (vision: Fig. 14, *top right*) was defined by:

(*A3*)
$$G(\alpha ,\nu )\;=\{\begin{array}{c} \kappa \cdot \nu \cdot \alpha^{2}\cdot e^{-(2a/\alpha_{\max})}\\ \text{with}\;k=6.1+7.3\;\cdot \;|\log\;(\nu /3){|}^{3}\\ \alpha_{\max}=45.9/(\nu +2) \end{array}$$

Here, α is the temporal frequency (Hz) and ν (cycles/octave) is the spatial frequency.

The parameters α_max_ and *k* are scaling factors. For details see Kelly (Ref. 45, p. 1345).

**Table A1.** Human and monkey auditory S-T MTF coefficients

Coefficient	Human MTF	Monkey MTF
*p00*	+7.196e−01	+6.527e−01
*p01*	+6.075e−03	+1.407e−02
*p02*	−1.453e−04	−2.436e−04
*p03*	+1.118e−06	+1.772e−06
*p04*	+3.862e−09	−5.977e−09
*p05*	+5.023e−12	+7.646e−12
*p10*	+6.676e−04	−6.000e−02
*p11*	−4.487e−04	+2.025e−04
*p12*	+2.994e−06	−6.307e−06
*p13*	+1.636e−08	+4.354e−08
*p14*	+3.416e−11	−8.366e−11
*p20*	−1.059e−02	−3.133e−02
*p21*	−9.817e−04	−1.051e−03
*p22*	+5.713e−06	+6.308e−06
*p23*	−1.092e−08	−1.173e−08
*p30*	−1.743e−04	+2.162e−02
*p31*	+5.116e−05	−2.110e−05
*p32*	−1.633e−07	+3.456e−08
*p40*	+1.017e−03	+2.486e−03
*p41*	+1.103e−05	+3.404e−06
*p50*	+1.096e−05	−1.662e−03

Each coefficient *p_nm_* belongs to polynomial term *x^n^y^m^* of *Eq. A2*, which includes terms up to order 5, under the constraint that 0 ≤ *n* + *m* ≤ 5. See glossary for abbreviations.
